# A Cross‐Sectional Survey of Evidence Needs for Medicinal Products in Europe With a Focus on Real‐World Evidence

**DOI:** 10.1002/pds.70358

**Published:** 2026-04-04

**Authors:** Maria Melinder, Fabian Windfuhr, Tanja Dahlqvist, Carla Torre, Bruno Sepodes, Peter G. M. Mol, Diogo Almeida, Isabella Ekheden, Nils Feltelius, Kit Roes, Sieta T. de Vries, Björn Wettermark

**Affiliations:** ^1^ Department of Pharmacy, Faculty of Pharmacy Uppsala University Uppsala Sweden; ^2^ Department of Clinical Pharmacy and Pharmacology University of Groningen, University Medical Center Groningen Groningen the Netherlands; ^3^ Faculdade de Farmácia Universidade de Lisboa Lisbon Portugal; ^4^ Laboratory of Systems Integration Pharmacology Clinical and Regulatory Science, Research Institute for Medicines of the University of Lisbon (iMED.ULisboa) Lisboa Portugal; ^5^ Dutch Medicines Evaluation Board Utrecht the Netherlands; ^6^ Department of Clinical Pharmacology Karolinska Institutet Solna Sweden; ^7^ Department of Public Health and Caring Sciences Uppsala University Uppsala Sweden; ^8^ Radboud University Medical Center Nijmegen the Netherlands; ^9^ Pharmacy and Pharmacology Center, Faculty of Medicine Vilnius University Vilnius Lithuania

**Keywords:** evidence needs, health technology assessment, real world evidence, regulatory decision‐making, regulatory science, RWE

## Abstract

**Purpose:**

To assess views among different stakeholders around evidence needs during assessment of clinical effects across the medicinal product life cycle, with a focus on real‐world evidence (RWE) in regulatory and health technology assessor (HTA)/payer decision‐making.

**Methods:**

A cross‐sectional survey was distributed between November 2023–January 2024 to 1210 European regulators, HTAs, payers, and other stakeholders (i.e., industry, academia, healthcare professionals, and patient representatives). The survey included questions on their experience and views on evidence needs, which were analysed descriptively and with non‐parametric testing.

**Results:**

191 respondents (110 regulators, 24 HTA/payers and 57 others) from 32 European countries completed the survey (response rate: 16%). Most respondents were between 41–60 years and 53% had > 10 years of work experience. Respondents were least confident in assessing evidence based on adjusted indirect comparisons and network meta‐analyses. Randomization and clinically relevant endpoints were considered most relevant in studies, while country‐specific data and patient preferences were considered least important. Respondents acknowledged that there are uncertainties on the clinical effect of medicinal products in the different stages of the medicinal product life cycle, but the views on the usefulness of RWE to address these uncertainties varied across stakeholder groups and life‐cycle stages. RWE was considered most likely to be accepted as supplementary evidence for orphan diseases and when randomized controlled trials (RCTs) are infeasible.

**Conclusion:**

Our findings highlight opportunities and challenges in using RWE to reduce uncertainties in decision‐making around medicinal products. This may contribute to a dialogue on how and when to augment evidence from RCTs with RWE.

## Summary

- A survey was sent out to different stakeholders involved in decision‐making around medicinal products in Europe, to assess views on different types of studies and evidence the stakeholders need for decision‐making. The focus was on so‐called real‐world evidence (RWE) – evidence based on data from routine clinical practice, as opposed to data from randomized controlled clinical trials (RCTs) which are usually considered the gold standard for assessing effect of a medicinal products. 191 persons responded to the survey (response rate: 16%), of whom 110 regulators, 24 health technology assessors/payers and 57 other stakeholders. We identified the study types that respondents were most and least confident in assessing, as well as which of the listed aspects of study designs (for example randomization) were considered most relevant for assessing effect. Respondents thought that there are uncertainties on clinical effect of medicines in several stages of the medicinal product life cycle, but the views on usefulness of RWE to address these uncertainties varied across stakeholder groups and life cycle stages. RWE was considered most likely to be accepted for rare diseases and when RCTs are not feasible. The findings highlight opportunities and challenges when using RWE to reduce uncertainties in decision‐making around new medicines.

## Key Points

1. This study shows views on evidence needs of regulators, health technology assessors (HTA), and payers, and other stakeholders when making decisions along the medicinal product life cycle.
2. Respondents were most confident about their skills in assessing evidence from randomized controlled trials (RCT) with real‐world data in planning, but least confident in assessing network‐meta analyses and adjusted indirect treatment comparisons.
3. Randomization, clinically relevant endpoints, and data quality were seen as the most important aspects of evidence, while country‐specific data and patient preferences were considered least relevant.
4. Evidentiary uncertainties were perceived to exist in all life‐cycle stages, but stakeholders had differing views on the usefulness of real‐world evidence (RWE) to address these uncertainties across life‐cycle stages.
5. Respondents were most likely to accept RWE as supplemental evidence in situations where it is infeasible to perform a traditional RCT, and for orphan drugs, but least likely for over‐the‐counter (OTC) medications and vaccines.

## 1 Introduction

Regulators, health technology assessors (HTAs), and other stakeholders need to make different decisions during the medicinal product life cycle based on the best available evidence [1]. Regulators focus on efficacy, safety, and the benefit–risk balance to assess whether a medicinal product should be granted market authorisation, while HTA is done to value the net benefit of a treatment compared to the current standard of care or alternate product [2]. HTA is often performed to inform decisions on resource allocation, for example, payer decisions on reimbursements [3]. Other stakeholders, such as healthcare professionals, patients, industry, and academia, make a wide range of different decisions as well, but are also affected by regulator, payer, and/or HTA decisions. Given the different decisions of stakeholders, their views on what evidence is needed to make those decisions may differ.

Randomized controlled trials (RCTs) are the gold standard for evidence on clinical effects of new medicinal products. However, they are often not large enough to detect rare side effects and the highly controlled environment can limit the generalizability to specific subgroups and patients in clinical practice [4]. Furthermore, new types of medicinal products, such as orphan medicines and advanced therapies, are challenging to study with traditional RCTs due to small target patient populations, high disease severity, and large unmet medical needs [5, 6]. This has led to an increased demand to complement evidence from RCTs with observational studies or “real‐world evidence” (RWE) for evaluation of benefit, risk and cost‐effectiveness [7, 8, 9]. RWE is defined by the European Medicines Agency (EMA) as evidence derived from the analysis of “real‐world data” (RWD) – data that describe patient characteristics (including treatment utilization and outcomes) in routine clinical practice [10]. New methods are being developed that may combine RCTs with RWE such as registry‐based RCTs, hybrid designs or indirect treatment comparisons incorporating RWE as an external control arm [11, 12, 13, 14]. Tools such as smart devices and big data analytics can also be used to better collect RWD from patients and healthcare professionals [15].

While criteria used to assess evidence by decision‐makers are often described in guidelines, how they are operationalized in specific scenarios can still be unclear [16]. Studies have shown that regulators and HTAs have both similarities and differences in their views on evidence and RWE [2, 17, 18, 19, 20]. However, surveys including both HTAs and regulators in the same study are sparse, as well as those including also other stakeholders affected by their decisions, despite efforts to harmonize demands from regulators and HTAs to reduce evidentiary uncertainties that could restrict patient access to medicinal products for which there is an unmet medical need [17, 21, 22].

The purpose of this study was therefore to investigate views on evidence needs of different stakeholders during assessment of clinical effects in the medicinal product life cycle, with a focus on RWE in regulatory and HTA/payer decision‐making.

## 2 Methods

### 2.1 Survey Design

A cross‐sectional web‐based survey study was conducted in the context of the More‐EUROPA project [23], a larger project focusing on more efficient use of RWD in the assessment of medicinal products. The survey was created and the data were collected and managed using REDCap (Research Electronic Data Capture; version 13.7.19) hosted at the University Medical Center Groningen [24, 25].

The survey was piloted among eleven regulators, HTA, payers, academia, and healthcare professionals and advisory board members within the More‐EUROPA project. Their feedback was incorporated into the final survey.

The survey consisted of 24 questions in English and included 4 sections: (1) general/demographic questions, (2) evidence needs, (3) RWE, and (4) patient registries. This study focuses on the first three sections. The results of the fourth section have been published elsewhere [20]. The general questions were worded slightly differently between regulators and HTAs/payers (Appendix A) and other stakeholders (Appendix B) to suit different backgrounds better.

The evidence needs section contained two questions: (1) “How confident are you that you have the knowledge and skills needed to assess evidence from the following study types?” for nine different study types, and (2) “How relevant do you consider the following aspects when assessing evidence on the clinical effects of a medicine?” with 14 methodological study aspects. These questions were assessed on 5‐point Likert scales. The section on RWE also had two questions: (1) “Across the different stages of the medicinal product life cycle (pre‐marketing, marketing authorization, post‐marketing, initial reimbursement and reassessment of reimbursement), are there remaining uncertainties regarding efficacy/effectiveness that could be addressed by using RWE?*”* with five answer options, and (2) “In your view, would any of the following aspects increase your likelihood to accept RWE as evidence on the clinical effect of a medicine?*”* (Appendix B) with three answer options for nine different scenarios chosen by the project team based on their previous experience of regulatory and HTA decision‐making. All questions were voluntary and included an “I don't know” answer option (for details see Appendices A and B).

### 2.2 Respondents and Data Collection

The study included European regulators, HTA, payers and other stakeholders (consisting of industry, patient representatives, healthcare professionals, academia) active in an area connected to pharmaceutical regulation and/or reimbursement. Consenting respondents ≥ 18 years and able to read and write in English were included. Details of the recruitment procedures have been reported previously [20]. In short, regulators were recruited through email invitations to members of various EMA committees and working parties. Email addresses were obtained through online search and prior correspondence and were then used to send a personal link to the online survey. Those who had not answered after 3 weeks received a reminder.

HTA/payers were recruited through contact persons in four networks, the PPRI network [26], RWE4Decisions [22], the Medicine Evaluation Committee (MEDEV) [27] and the European Network for HTA (EUNetHTA) [28]. The contact persons disseminated the survey invitation to members and/or contacts within the networks (thus not sharing personal information with the research team) and reported back the number of invitees—enabling calculation of a response rate for the survey.

In addition, registrants of a webinar on the use of registries in regulatory decision‐making, organized by the More‐EUROPA consortium [29], were invited by email with a personal survey link. The 479 registrants included regulators and HTA/payers, but also other stakeholders such as industry, academics, patient representatives, and registry holders.

### 2.3 Data Analysis

Respondents who answered at least one content‐related question (any question outside of section 1: general/demographics) were included in the analyses and grouped into one of the following three clusters based on their professional roles: (1) Regulators, (2) HTA/payers and (3) Other stakeholders (“Others”). Respondents could choose multiple organizations that they worked for, but in the analysis, they were only included in one of the three groups. All who worked for an HTA/payer organization were included in the HTA/payer group, regardless of other affiliation. Subsequently, whoever indicated that they worked for a regulatory agency (unless they were also an HTA/payer) was included in the regulator group. The remaining respondents, including those who did not state any organization, were included in the “Others” group.

Responses were analysed descriptively in Microsoft Excel (version 2018), pooling all respondents, as well as stratified by stakeholder group. For all questions, the absolute and relative frequency distribution for each answer option was graphically presented in diverging bar charts. The number of respondents per group that did not answer the question or chose the “I don't know” option was also summarized. In addition, for responses to the questions with 5‐point Likert scales, median, mean, and 95% confidence interval (CI) based on sample standard deviation were calculated in R (version 4.3.2) [30] to summarize the distribution of responses. To compare answers between stakeholder groups, Kruskal Wallis tests and post hoc pairwise comparisons (Dunn's) with Bonferroni correction for multiple testing were performed on a 5% significance level. Respondents with missing data (those who selected “I don't know” or left the question blank) were excluded from these analyses.

## 3 Results

Of 1210 persons invited, 263 persons (22%) consented to participate. Of these, 68 did not respond to any content‐related questions and were therefore excluded. Further, four persons were excluded based on being outside of Europe. Of the 191 included respondents (response rate: 16% of all invitees), most were regulators (*n* = 110); 24 indicated they worked as HTA, as payers or both, and there were 57 respondents in the Others group. The respondents' characteristics are presented in Table 1.

**TABLE 1:** Respondents' characteristics overall and by stakeholder group.

		Overall	Regulators	HTA/Payers	Others
Total number of respondents		191	110	24	57*
**Age**	*18–30 years*	16 (8.4%)	3 (2.7%)	4 (16.7%)	9 (15.8%)
	*31–40 years*	40 (20.9%)	21 (19.1%)	6 (25.0%)	13 (22.8%)
	*41–50 years*	62 (32.5%)	33 (30.0%)	8 (33.3%)	21 (36.8%)
	*51–60 years*	47 (24.6%)	33 (30.0%)	6 (25.0%)	8 (14.0%)
	*61–70 years*	21 (11.0%)	16 (14.5%)	0	5 (8.8%)
	*71+ years*	2 (1.0%)	1 (0.9%)	0	1 (1.8%)
	*I prefer not to answer*	3 (1.6%)	3 (2.7%)	0	0
**Gender**	*Woman*	124 (65.5%)	72 (65.5%)	14 (58.3%)	38 (66.7%)
	*Man*	65 (32.7%)	36 (32.7%)	10 (41.7%)	19 (33.3%)
	*Other*	0	0	0	0
	*I prefer not to answer*	2 (1.0%)	2 (1.8%)	0	0
**Years of experience in this field or organization**	*0–5 years*	55 (28.8%)	29 (26.4%)	7 (29.2%)	19 (33.3%)
	*6–10 years*	34 (17.8%)	16 (14.5%)	4 (16.7%)	14 (24.6%)
	*> 10 years*	101 (52.9%)	64 (58.2%)	13 (54.2%)	24 (42.1%)
	*I prefer not to answer*	1 (0.5%)	1 (0.9%)	0	0

* The 57 other stakeholders consisted of 25 industry, 7 academia, 3 healthcare professionals, 4 patient representatives, 9 people who had different combinations of these roles, 6 who stated “Other organization” and 3 respondents who did not choose any organization.

The respondents worked for organizations originating in 32 European countries (Figure S1). A total of 26 respondents (14%) stated that they work for multinational organizations, such as the EMA or pharmaceutical companies. Among those not working for multinational organizations, the country with most respondents was the Netherlands. Most regulators worked with initial marketing authorizations (80%) and scientific advice (64%). Most HTA/payers worked with reimbursement (63% with initial reimbursement, and 50% with reimbursement reassessment); a third of this group also worked with scientific advice. Of the Others, 44% were involved in post‐marketing research (e.g., pharmacovigilance and pharmacoepidemiology). Of the respondents, 49% worked within all therapeutic areas or did not focus on any specific therapeutic area. The most common specific area was oncology and/or radiopharmaceuticals (*n* = 45). A full table of areas of involvement for respondents is found in Table S2.

### 3.1 Confidence in Assessing Results From Different Study Types

**FIGURE 1:**
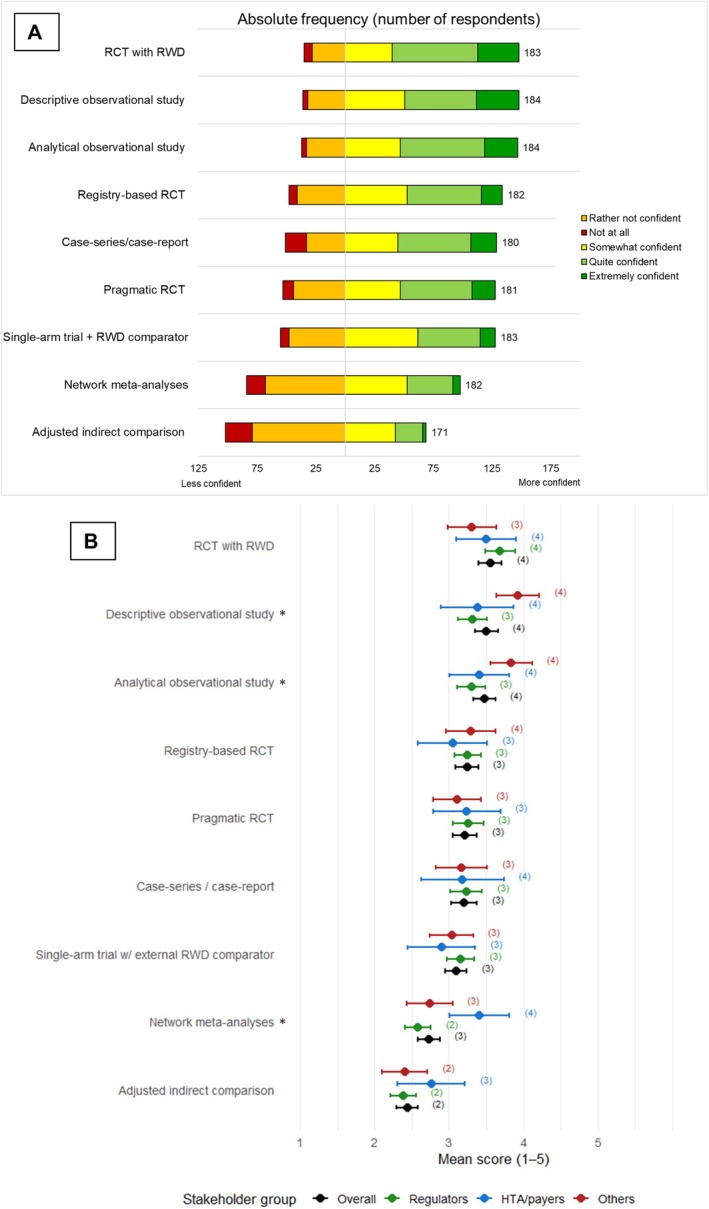
(A) Distribution of responses for “having the knowledge and skills needed to assess evidence from the following study types” on a scale from 1 not at all to 5 extremely confident, in absolute frequencies including the total number of respondents who chose one of the five answer options (i.e., did not choose “I don't know” or skip the question). (B) Means with 95% confidence intervals and corresponding median in brackets for respondents' stated confidence in knowledge and skills to assess evidence from different study types on a Likert scale (1 Not at all confident to 5 Extremely confident). The different study types are presented in order of highest point mean by all respondents at the top to lowest at the bottom. * indicates there was a *p* < 0.05 in the Kruskal Wallis test between stakeholder groups. Details on number of respondents per answer option, including “I don't know” and missing responses is presented in Tables S3–S6. RCT = randomized controlled trial; RWD = real‐world data; HTA = health technology assessors.

Overall, respondents were confident in assessing evidence from most of the study types (Figure 1A). They were least confident in assessing adjusted indirect comparisons (median 2 = rather not confident, mean 2.73, 95% CI 2.53–2.92), while most confident assessing RCTs with RWD in planning (median 4 = quite confident; mean 3.55, 95% CI 3.39–3.71 and descriptive and analytical observational studies respectively, median 4; mean 3.51, 95% CI 3.35–3.66 and median 4; mean 3.47, 95% CI 3.32–3.62, Figure 1A). For all three groups, adjusted indirect comparisons also had the most missing and/or “I don't know”, details in Tables S3 and S4.

Statistically significant differences across the stakeholder groups were found for three of the nine assessed study types, i.e., descriptive and analytical observational studies and network meta‐analysis (Figure 1B and Table S5). More specifically, the Others group expressed higher confidence than regulators for both descriptive and analytical observational studies (*p* = 0.002 and *p* = 0.007 respectively). HTA/payers had higher confidence than both regulators and Others in assessing Network meta‐analyses (*p* < 0.001 and *p* = 0.013 respectively).

### 3.2 Perceived Relevance of Different Study Aspects in Evidence Assessment

**FIGURE 2:**
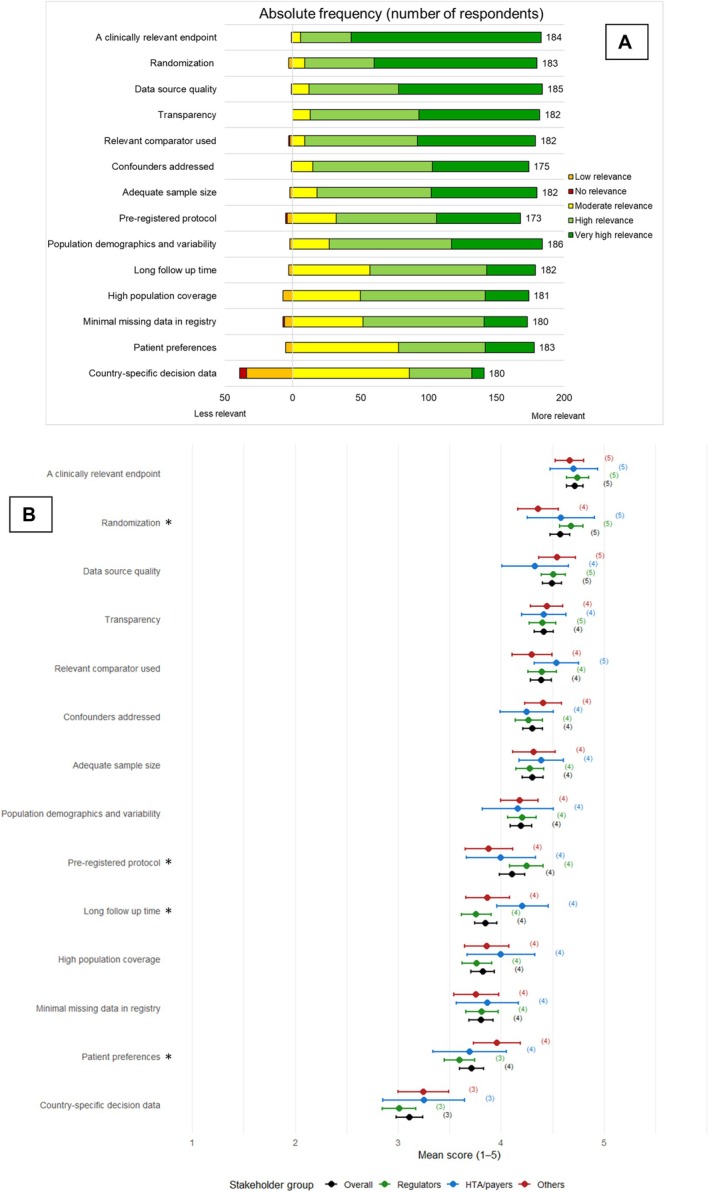
(A) Distribution of responses regarding how relevant the study aspects were for assessing evidence on clinical effect of a medicine on a scale of 1, No relevance to 5, Very high relevance, presented as absolute frequency of all respondents including the total number of respondents who chose one of the five answer options (i.e., did not choose “I don't know” or skip the question). Instead of “medicinal product”, the more colloquial term “medicine” was used in the survey. (B): Means with 95% confidence intervals (CI) for responses in total (all) and per group regarding how relevant the study aspects were for assessing evidence on clinical effect of a medicine on a scale of 1, No relevance to 5, Very high relevance. The study aspects are presented in order of highest point mean by all respondents at the top to lowest at the bottom in both figures. “*” indicates that there was a *p* < 0.05 in the Kruskal Wallis test between stakeholder groups, details in Tables S4 and S5. HTA = health technology assessors. The number of respondents per answer option in total and per group including “I don't know” and missing responses for the assessed study aspects is presented in Table S8 and Figure S9.

In general, all presented study aspects were considered highly relevant by the respondents, except for country‐specific decision data which was considered moderately relevant (median 3, mean 3.11, 95% CI: 2.98–3.24, Figure 2A). The most relevant aspects were a clinically relevant endpoint (median 5, mean 4.72, 95% CI: 4.64–4.80) and randomization (median 5, mean 4.57, 95% CI: 4.48–4.67).

The perceived relevance of the different aspects of study designs was generally similar across the stakeholder groups (Figure 2B, details in Files S4–S6). A statistically significant difference across stakeholder groups was shown for randomization, pre‐registered protocol, patient preferences, and long follow‐up time. Regulators considered randomization and pre‐registered protocols more relevant than the Others (*p* = 0.004 and *p* = 0.015, respectively), whereas the Others considered patient preferences more relevant than regulators (*p* = 0.035; Figure 2B). Furthermore, HTA/payers considered long follow up time more relevant than regulators (*p* = 0.023).

### 3.3 Opinions on Uncertainties During the Medicinal Product Life Cycle That Could Be Addressed by RWE

**FIGURE 3:**
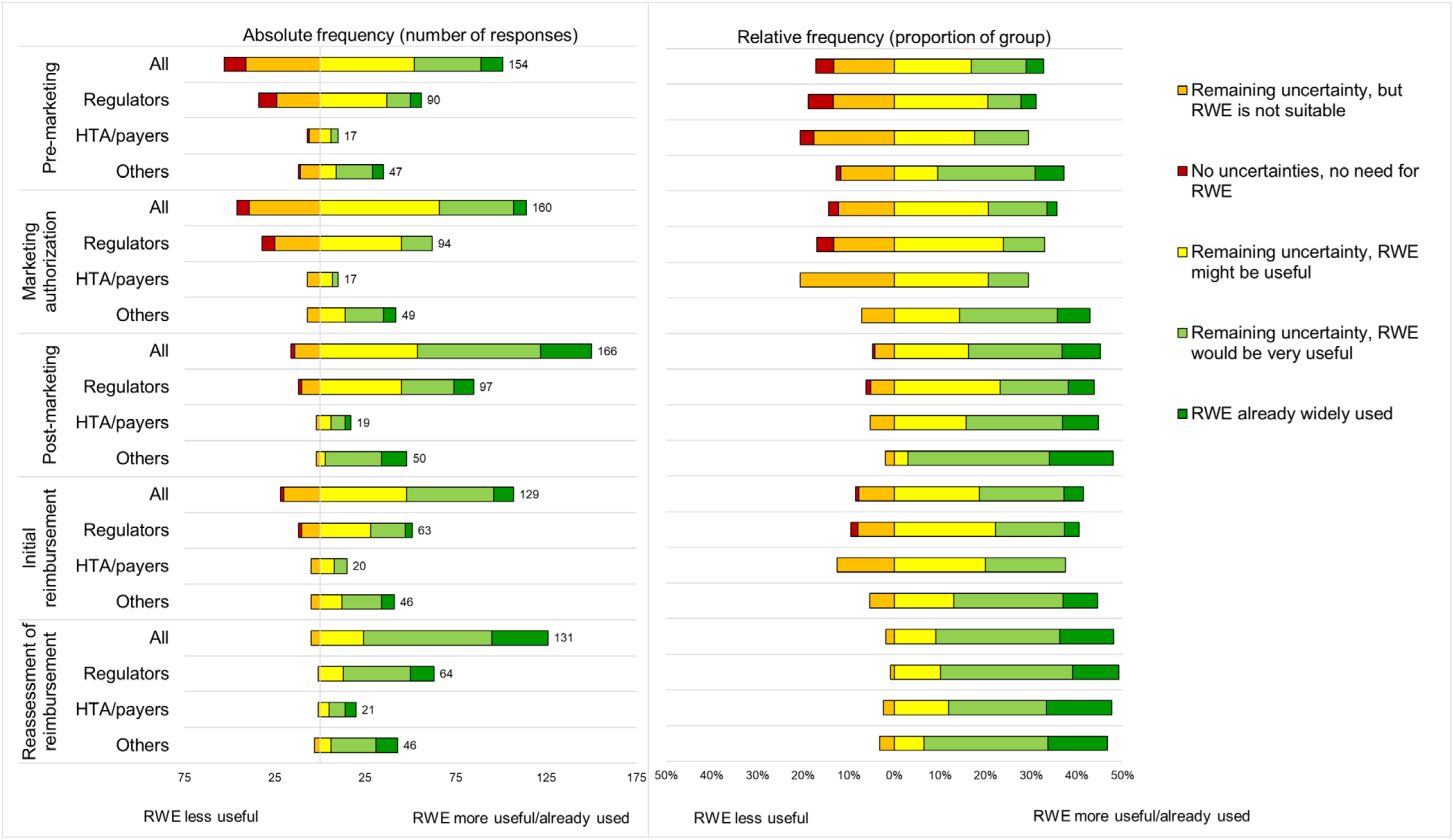
Respondents views on remaining uncertainties regarding efficacy/effectiveness that could be addressed by using RWE in different medicinal product life‐cycle stages in relative and absolute frequencies, including the total number of respondents who chose one of the five answer options in each group. Between 12% and 42% of respondents across groups and stages stated they did not know or left this question blank. The number of respondents per answer option, including “I don't know” and missing responses per group is presented in Table S10.

Respondents indicated that there are uncertainties regarding efficacy/effectiveness in all the mentioned life‐cycle stages. RWE was viewed as most useful or already widely used in the reimbursement and post‐marketing stages, while still potentially useful in the pre‐marketing and marketing authorization stages (Figure 3).

However, the opinions on whether RWE would be useful were slightly different across stakeholder groups. Of those who chose another option than “I don't know”, the Others group had a higher proportion than regulators and HTA/payers of responses that RWE either could be useful or was already widely used in all stages except for reassessment of reimbursement (Figure 3). In the initial reimbursement stage, 25% of responding HTA/payers stated there are no uncertainties or RWE is not suitable as compared to 11% and 19% for Others and Regulators, respectively.

### 3.4 Views on RWE in Certain Situations

**FIGURE 4:**
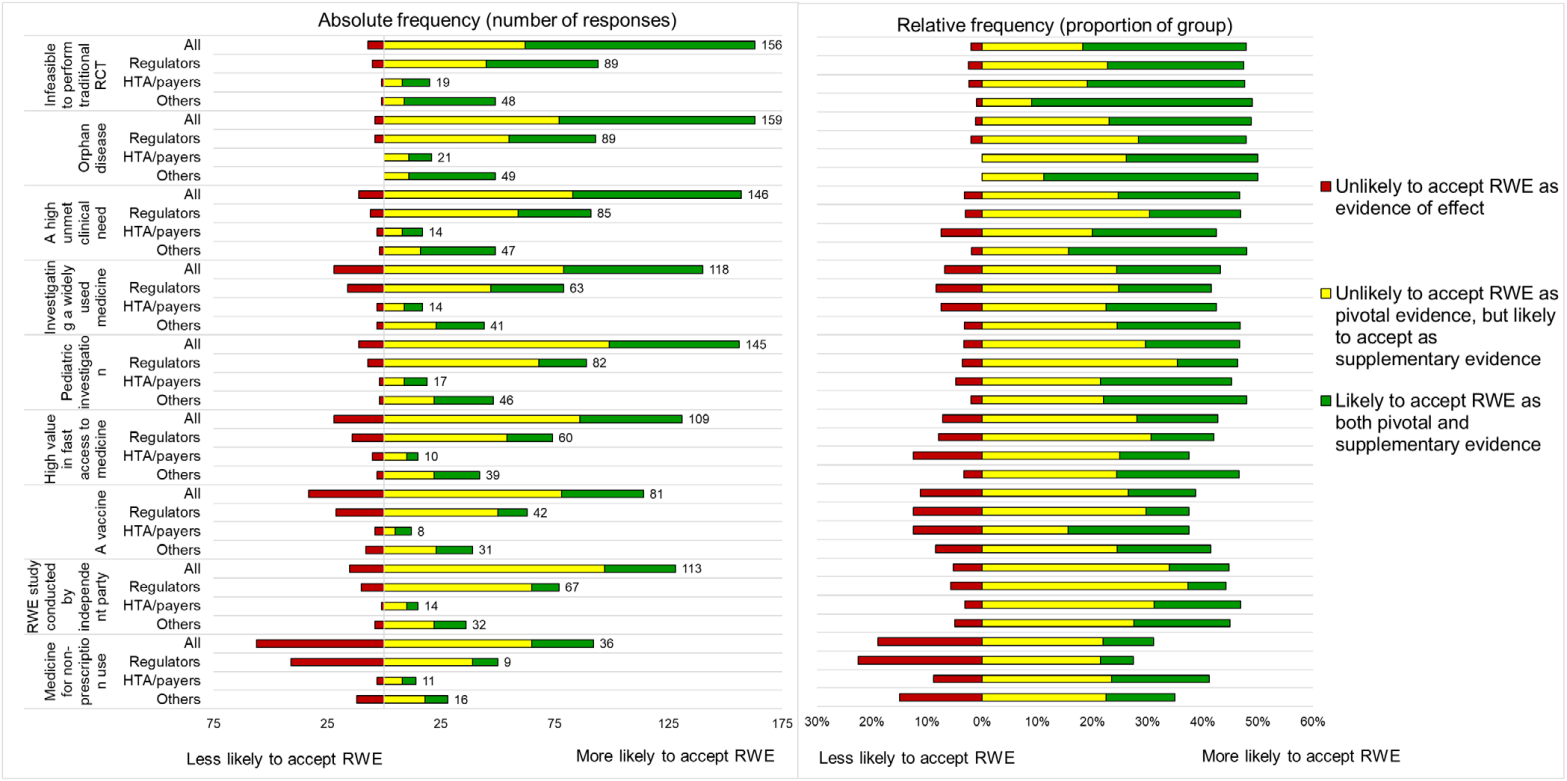
Respondents' views on likelihood of accepting RWE as evidence of the clinical effects of medicines in different situations in relative and absolute frequencies, including the total number of respondents who chose one of the five answer options in each group. RCT = randomized controlled trial; RWE = real‐world evidence. The number of respondents per answer option, including “I don't know” and missing responses per group is presented in Supporting Information Table S11.

Across all respondents, RWE was most likely to be accepted as both supplementary and pivotal evidence for clinical effect in situations where it is not feasible to perform a traditional RCT (53%), in the context of an orphan disease (45%), and when there is a high unmet clinical need (39%; Figure 4). For vaccines, non‐prescription medicines, and situations where a RWD study is conducted by an independent party, at least 20% of respondents did not know or left this question blank. This pattern was similar for all three stakeholder groups (Table S11). Of those who chose another option than not responding or selecting “I don't know”, the Others had a higher proportion of accepting RWE as both supplemental and pivotal evidence of effect compared to HTA/payers and regulators in all situations except for medicines for non‐prescription use and vaccines (Figure 4).

## 4 Discussion

This study addresses views on evidence needs among regulators, people working in HTA bodies, payers, and other stakeholders around introduction and decisions along the life cycle of medicinal products.

The respondents were confident in assessing most of the study types. They were least confident in assessing adjusted indirect analyses, and the regulators and Others groups were also less confident than HTA/payers in network‐meta analyses, highlighting the need for training and educational efforts on these methods. The Others group was more confident than regulators in assessing evidence from descriptive and analytical studies. The Others group also tended to be more likely than regulators to choose alternatives where RWE would be useful or is already widely used in most life cycle stages, as well as being more accepting of RWE as evidence in several situations. This could indicate that the Others group in this study had a more positive view of RWE than the regulators. The Others group did include several stakeholders (mainly industry, but also academia and patient representatives, for example) that are not accountable for the decisions of approval and reimbursement of medicinal products in the same way as regulators and payers are, which could influence this finding. However, the diversity of stakeholders within the Others group and the recruitment through the registry webinar may also affect these results, see limitations section below.

The respondents considered clinically relevant endpoints, randomization, and high data quality to be the most relevant for assessment of clinical effect. A recent study observed that both regulators and HTAs value long‐term outcomes but that they are more important to HTAs [17]. Our study indicated a similar tendency that a long follow‐up time might be seen as more relevant by HTA/payers than regulators. Jansen et al. also found that the most important differences between regulators and HTAs were in choice of comparator, preferred endpoints, and target populations [17]. In our study, no statistically significant differences between regulators and HTA/payers were observed regarding comparator or clinically relevant endpoints. Our study adds to previous research as it indicates that the Others were more positive toward the relevance of patient preferences than regulators, while regulators indicated higher relevance of randomization and pre‐registered protocols than the Others group. However, the relevance of study aspects and view on usefulness of RWE depend heavily on the specific context of different cases—which other treatments are available, the disease prevalence and severity, cultural differences, and many other factors [2, 17, 18]. Such details were not specified in our survey but should be considered in future studies.

The evolving landscape of drug development, with a trend towards treatments targeted at rare diseases, or small subpopulations, results in an increasing number of conditional approvals and increasing uncertainties, especially for downstream HTA and reimbursement decision‐makers [21, 31]. Respondents generally indicated that there are uncertainties regarding the efficacy and/or effectiveness in most stages of the medicinal product life cycle and that there is at least potential usefulness of RWE but that it is not often already widely used. While this question had a large proportion of participants who did not know or left the question blank—increasing the uncertainty of the results—the findings regarding current use are mirrored by previous regulator assessments. While 40% of marketing authorisation applications to EMA between 2018 and 2019 contained references to RWD/RWE, it was only considered to support decision‐making with evidence on clinical effects in a few of those cases [32]. Regulators have previously shown higher willingness than HTA to accept new types of evidence [2]. HTA/payers have expressed concerns around the current lack of comparative studies at the time of market authorization and that this will be further exacerbated with the review of the EU medicinal product legislation [33]. Although this study only includes a small sample of HTA/payers, it is noteworthy that the views of the responding regulators and the HTA/payers were largely similar. Respondents in general were likely to accept RWE as supplementary evidence in several situations, even as both supplementary and pivotal evidence in some contexts—mostly for orphan diseases and when it is infeasible to perform a traditional RCT, consistent with previous findings [22, 34]. A survey of HTA organizations found similar results, where RWD was most likely to be accepted in orphan diseases or other small populations [35]. In our study, regulators and HTA/payers more seldomly stated their views about uncertainties and/or usefulness of RWE for life‐cycle stages which they do not make decisions for. A lack of understanding of evidence needs has previously been identified as a barrier to aligning procedures and fulfilling the evidence needs of both regulators and HTAs [2, 17]. Our results are in line with this and indicate that collaborations and educational efforts could address this gap.

The views on usefulness of RWE identified in this study align with broader regulatory frameworks and initiatives that have begun to incorporate RWE more extensively, and where the acceptability of evidence is multifaceted and influenced by the decision context, treatment alternatives, unmet needs, methodological aspects and price‐related aspects among others [22, 34, 35, 36]. Several previous studies highlight the importance of collaboration and transparency among stakeholders throughout the medicinal product life cycle, especially early on [22, 37, 38]. Uncertainties in EMA decisions have been linked to negative reimbursement decisions, limiting patient access to these medicinal products [21]. For example, by aligning evidence requirements where possible and with joint preapproval advice procedures evidentiary uncertainties can be reduced for later HTA and payer decisions [17, 22, 38]. This also extends to industry developing medicinal products. Our survey results could be useful in these cross‐stakeholder collaborations and discussions.

However, further studies are needed to delve deeper into potential differences in needs between stakeholders with study designs more suitable for causal inference and exploring the stakeholders in the Others group further. In particular, the relative perceived relevance of different study aspects assessed in this study could be used to inform further stated preference studies in situations where RWE has been identified as potentially useful, as have been done for example on payers in the United States [39]. Views on in what particular situations it is infeasible to perform a RCT and what role RWE could play could also be explored further in qualitative studies. The ever‐developing legislative environment around medicinal products, especially with new legislation also around the European Health Data Space [40] and increased international cooperation through the new European HTA regulation [41] may influence views on RWE and evidence needs. Therefore, it would be relevant to follow these views over time to also assess the impact of these new initiatives.

### 4.1 Strengths and Limitations

A strength of this study is the inclusion of several different stakeholder groups in the same survey to enable comparisons. The study also benefits from a wide geographical spread improving generalizability and relevance to the broader European context. The low response rate is a limitation. The respondents could be more predisposed to using RWE compared to the source population because of self‐selection and the distribution method, especially for those who were recruited through the registry webinar, which should be considered when interpreting the results. Since the More‐EUROPA project has a focus on regulator and HTA decision‐making, the respondents were divided into Regulators, HTA/payers and Others [23]. However, the Others were a diverse group of stakeholders which, if examined in a survey with more respondents, could have been stratified further to better reflect these groups. The regulator group was larger, but the Others and especially the HTA/payer groups were smaller making these results more uncertain. For the HTA/payers, invitations were sent out through specific networks, resulting in the very small sample size. The responses should not be seen as generalizable to the larger community of HTA/payers in Europe nor the responses in the Others group representative for all other stakeholders. Any potential differences between groups should be interpreted with caution and explored in further studies. On the other hand, the recruitment approach for HTA/payers allowed for control of the response rate and ensured distribution to a variety of countries. Another limitation was the relatively extensive survey which may introduce response fatigue, although this would apply less to the questions used in this study as they were placed earlier on in the survey.

## 5 Conclusions

This study shows perceived evidence needs of regulators, HTAs, payers, and other stakeholders along the life cycle of medicinal products, contributing to the ongoing discussion on the role of RWE in supplementing traditional RCTs. The respondents considered aspects of RCTs important (e.g., randomization) while the findings also showcase the perceived usefulness of RWE in several life‐cycle stages and situations. Similarities but also potential differences across stakeholder groups and life‐cycle stages have been identified. This may contribute to a dialogue and inform further studies on how and when to augment evidence from RCTs with RWE to reduce uncertainties in decision‐making around medicinal products.

## Funding

This project has received funding from the European Union's Horizon Europe Research and Innovation Actions under grant no. 101095479 (More‐EUROPA). Views and opinions expressed are however those of the author(s) only and do not necessarily reflect those of the European Union nor the granting authority. Neither the European Union nor the granting authority can be held responsible for them. The results were presented in a poster during the International Society of Pharmacoepidemiology (ISPE) Annual Meeting in Berlin in August 2024.

## Ethics Statement

For this study, we conducted a cross‐sectional, anonymous survey. The study protocol has been assessed by the Swedish Ethical Review Authority receiving a waiver from ethical review (ref no: 2023–07187‐01). A waiver for full ethical approval was also obtained after evaluation by the Medical Ethics Review Board of the University Medical Center Groningen (METc UMCG; ref. no: M23.323558).

## Conflicts of Interest

The authors declare no conflicts of interest.

## Supporting information




**Appendix A.** Regulator and HTA/payer version of the survey.
**Appendix B**. Survey used for other stakeholders than regulator and HTA/payers.
**Appendix C**. Supplementary figures and tables.
