# Supplementary material for: A Cross‐Sectional Survey of Evidence Needs for Medicinal Products in Europe With a Focus on Real‐World Evidence
Source: Pharmacoepidemiol Drug Saf. 2026 Apr 4;35(4):e70358. doi: 10.1002/pds.70358 (PMC13049559; doi:10.1002/pds.70358)
Supplement: Supplementary file 1 — Appendix A. Regulator and HTA/payer version of the survey. Appendix B. Survey used for other stakeholders than regulator and HTA/payers. Appendix C. Supplementary figures and tables. [file PDS-35-e70358-s001.docx]

Supplementary material

## Appendix A – Regulator and HTA/payer version of the survey


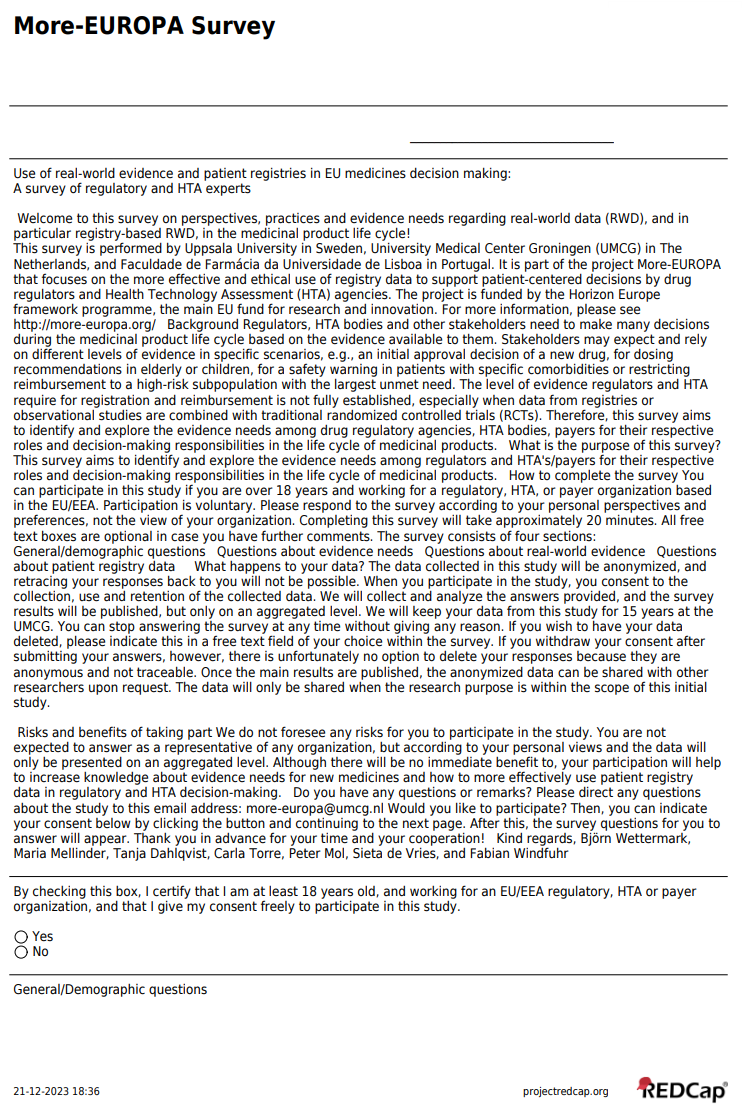


**
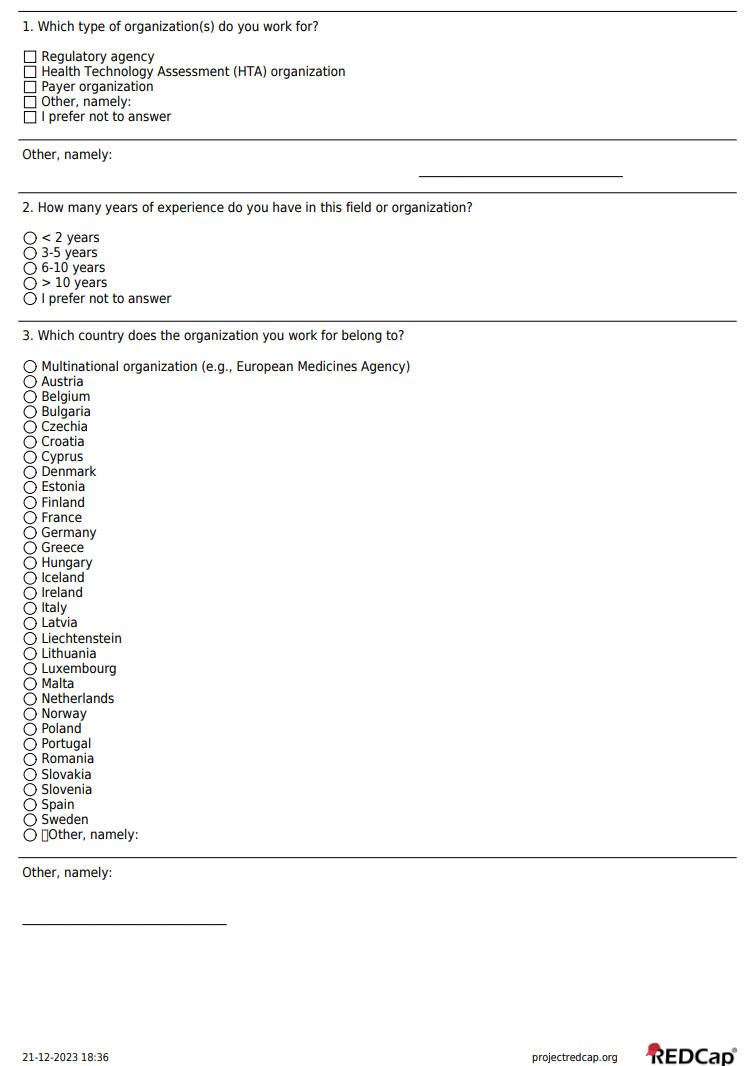
**

**
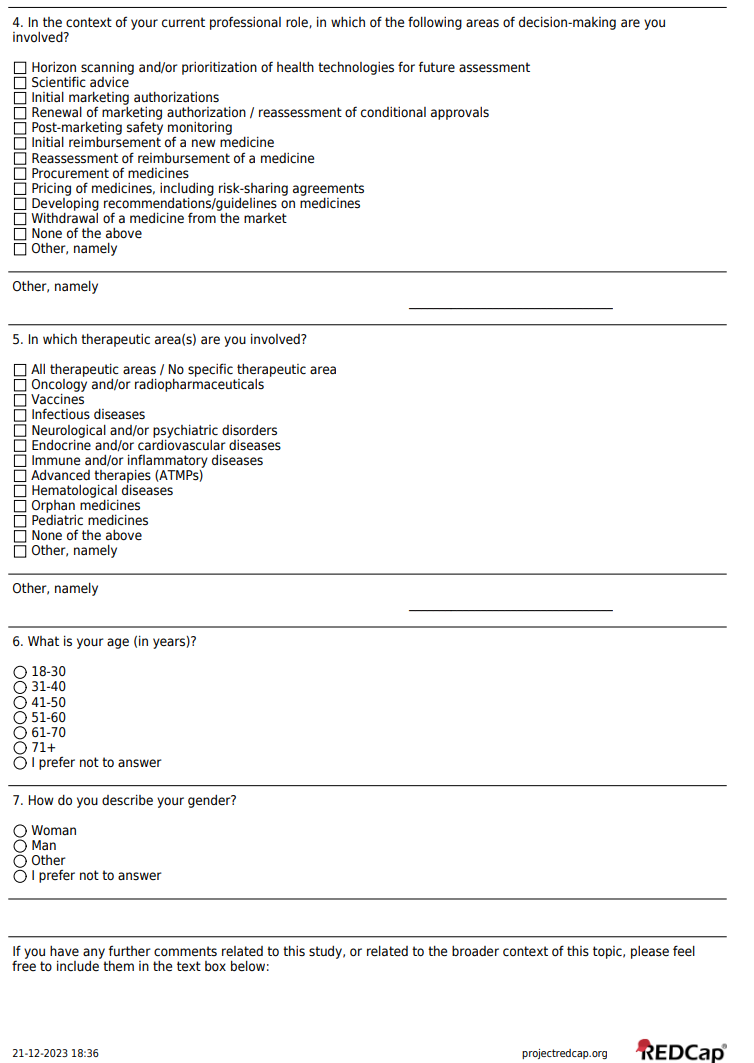
**

**
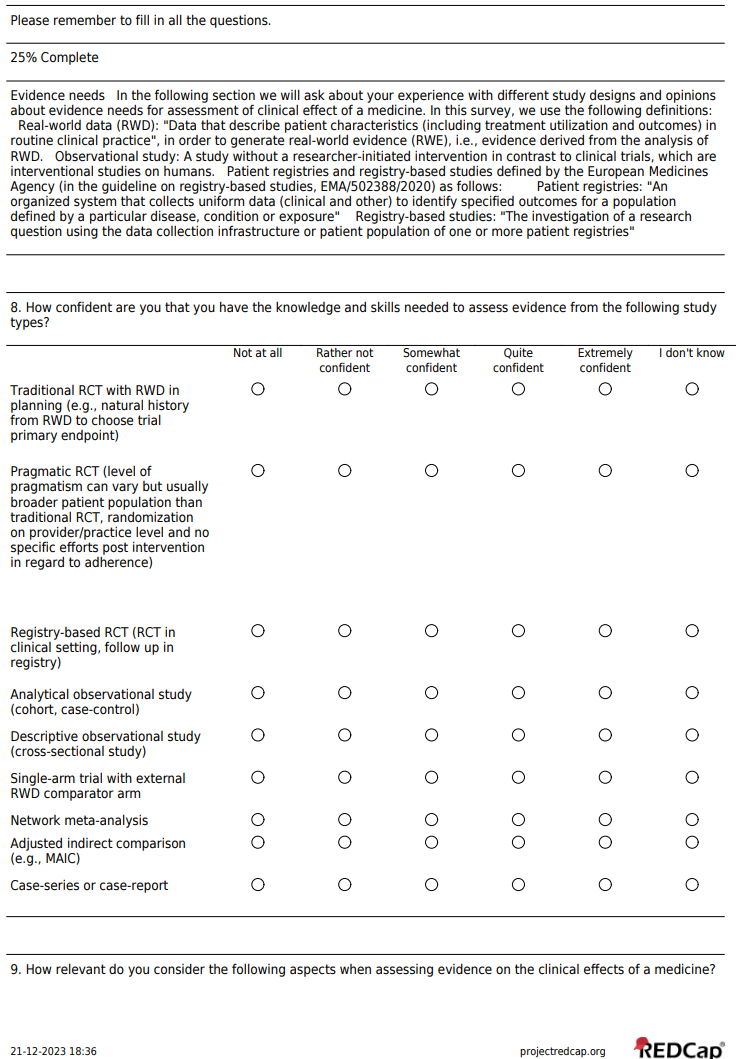
**

**
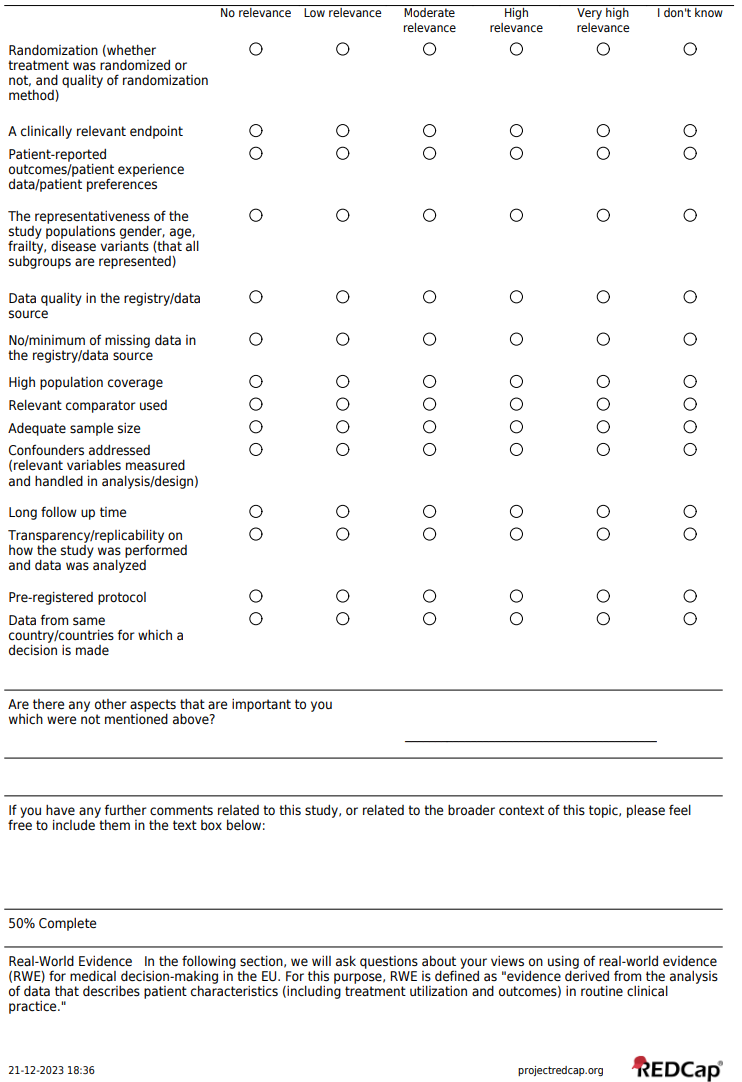
**

**
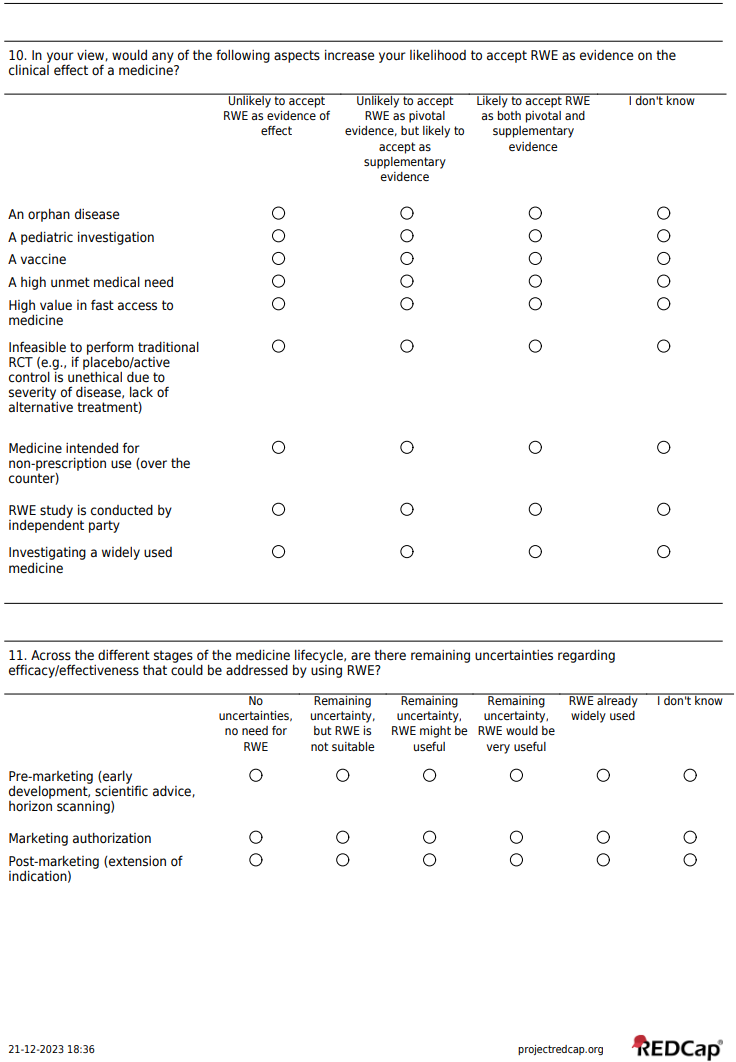
**

**
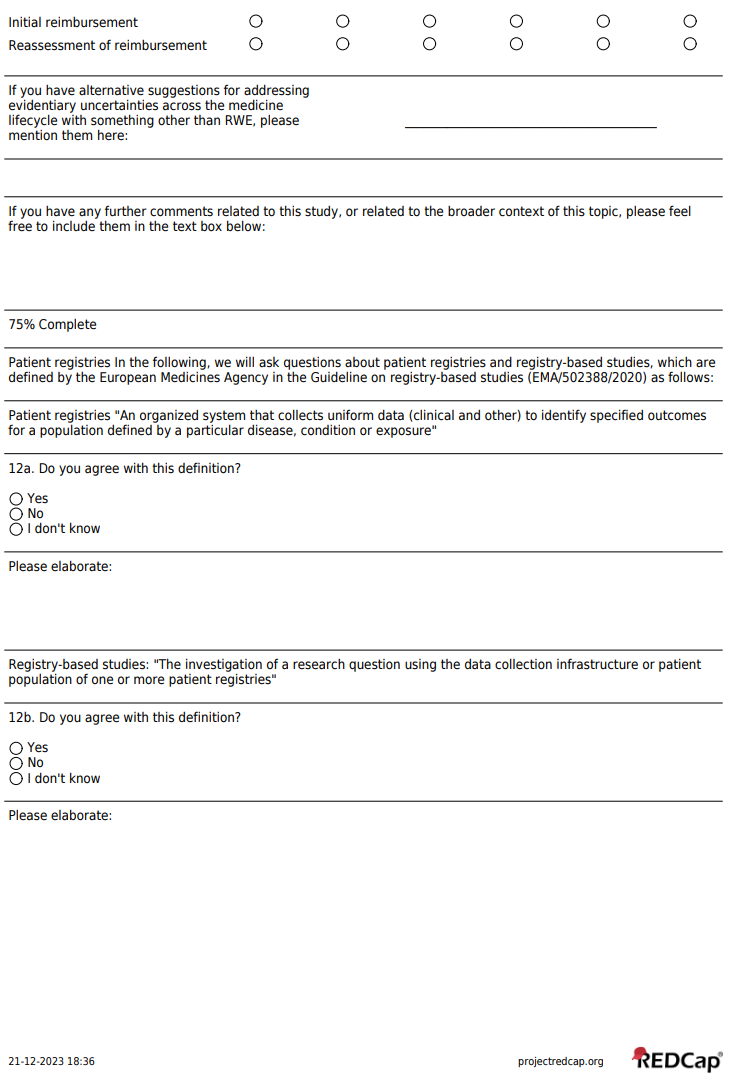
**

**
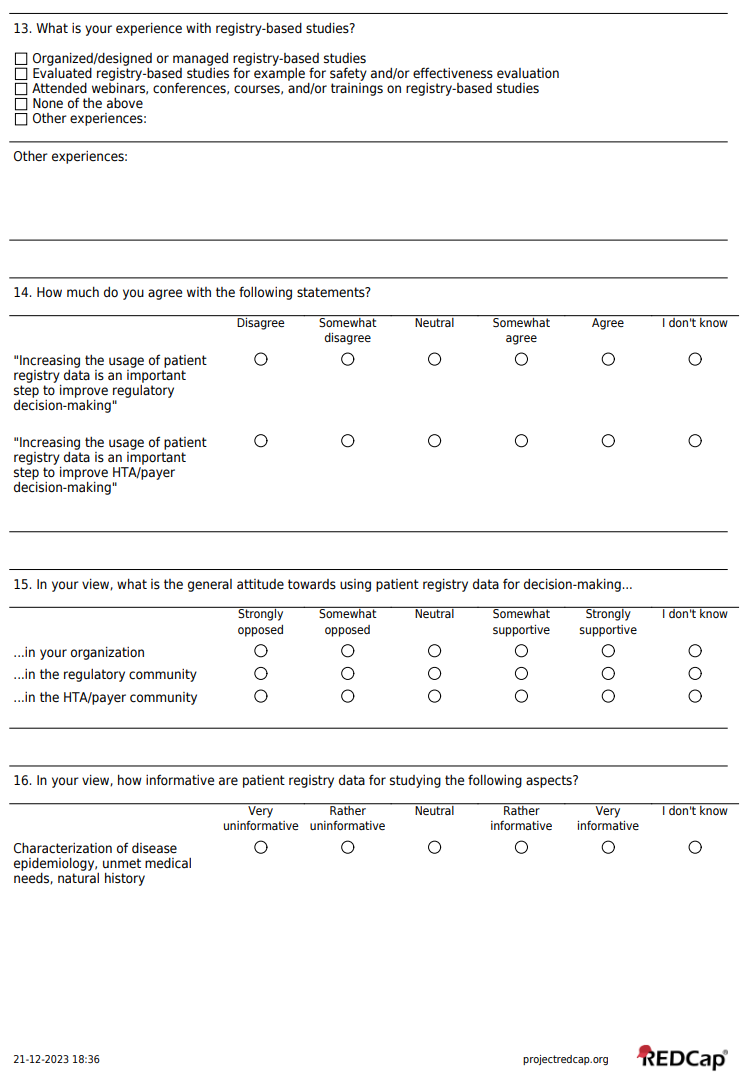
** **
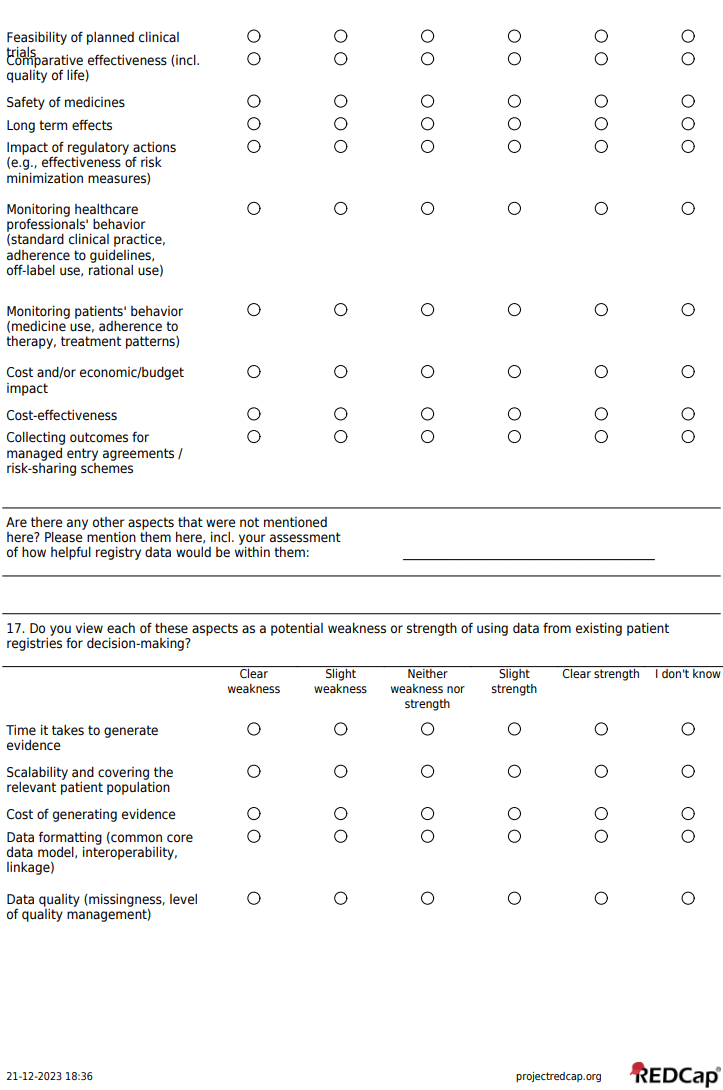
**

**
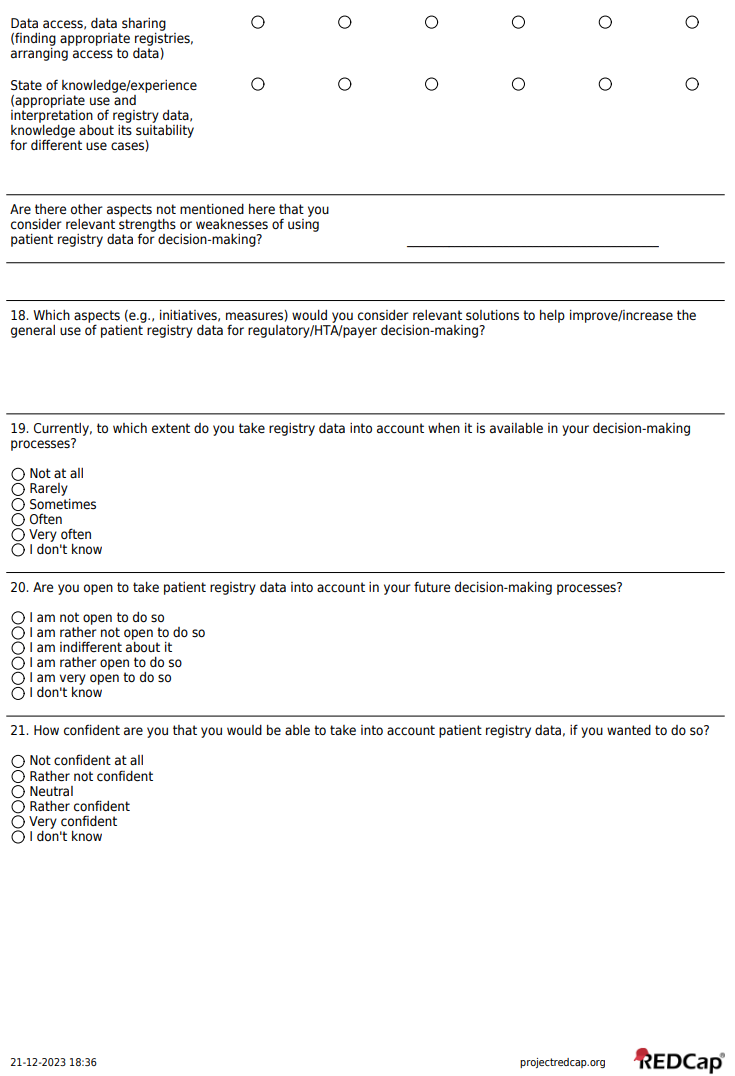
** **
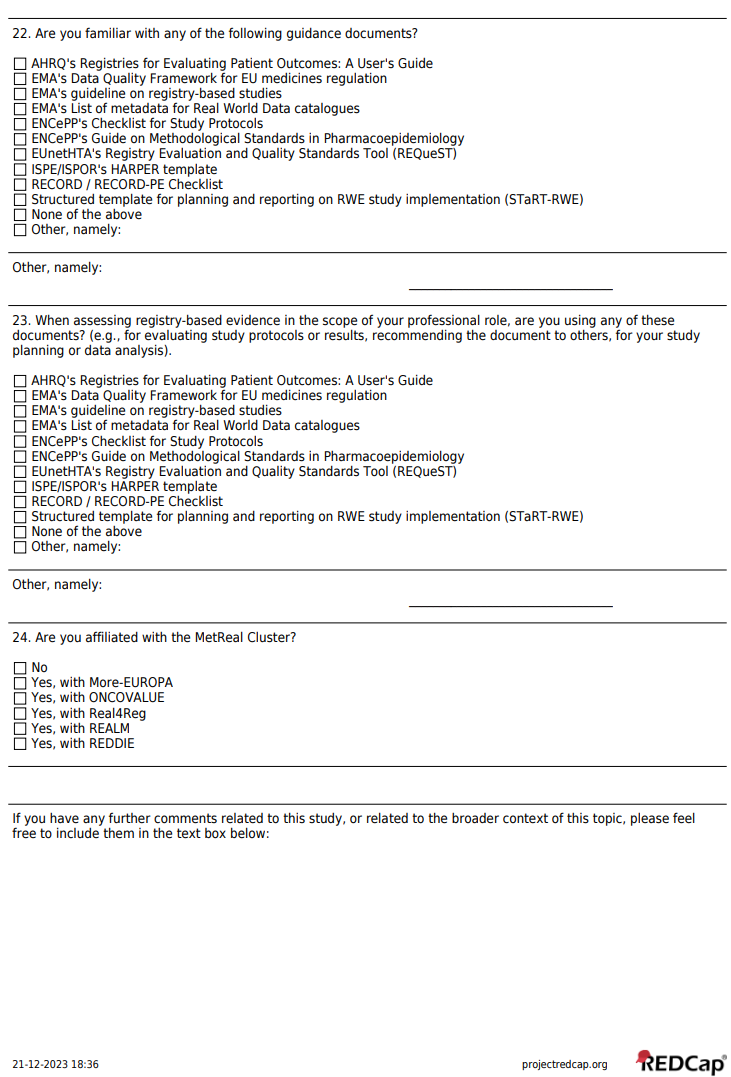
**

## Appendix B – Survey used for other stakeholders than regulator and HTA/payers


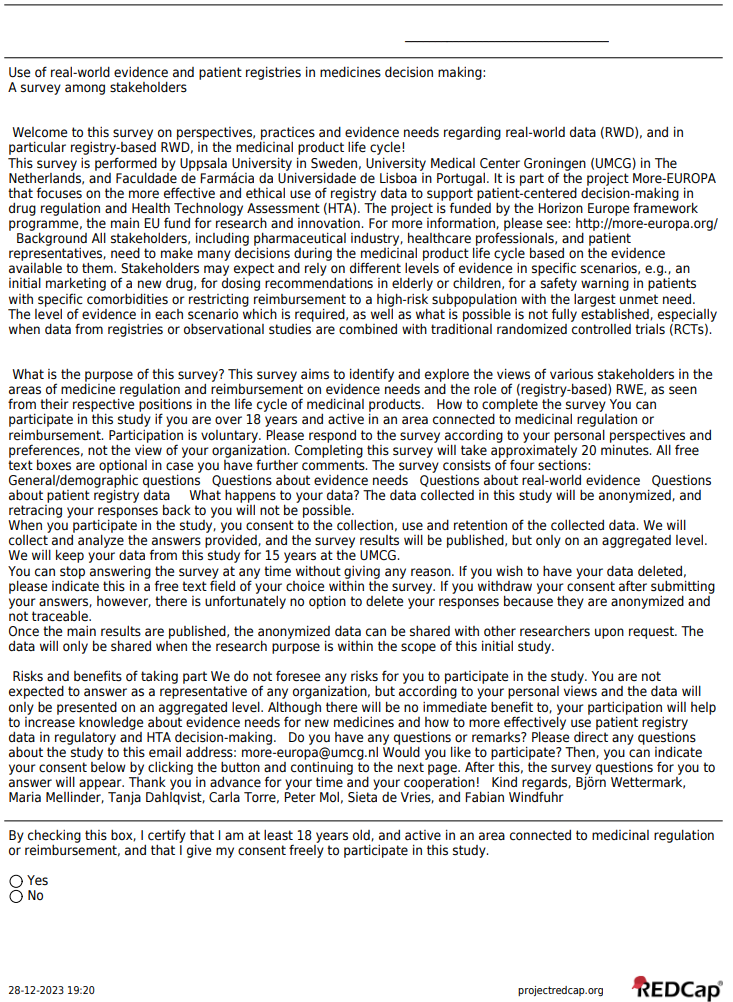


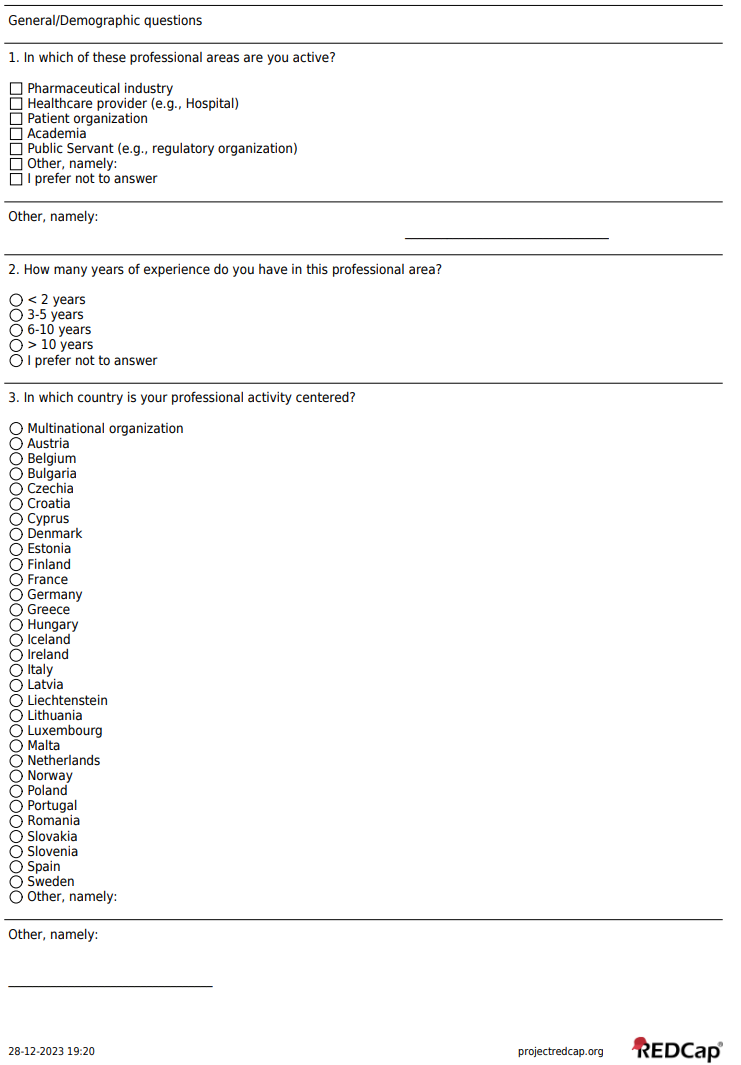


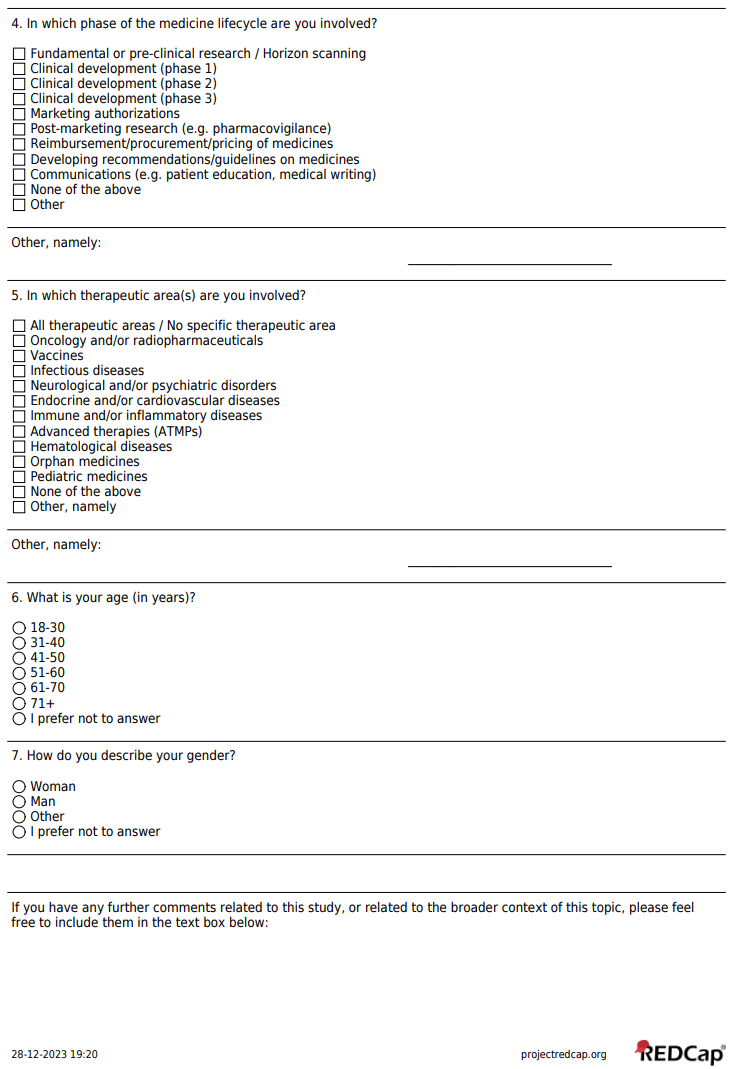

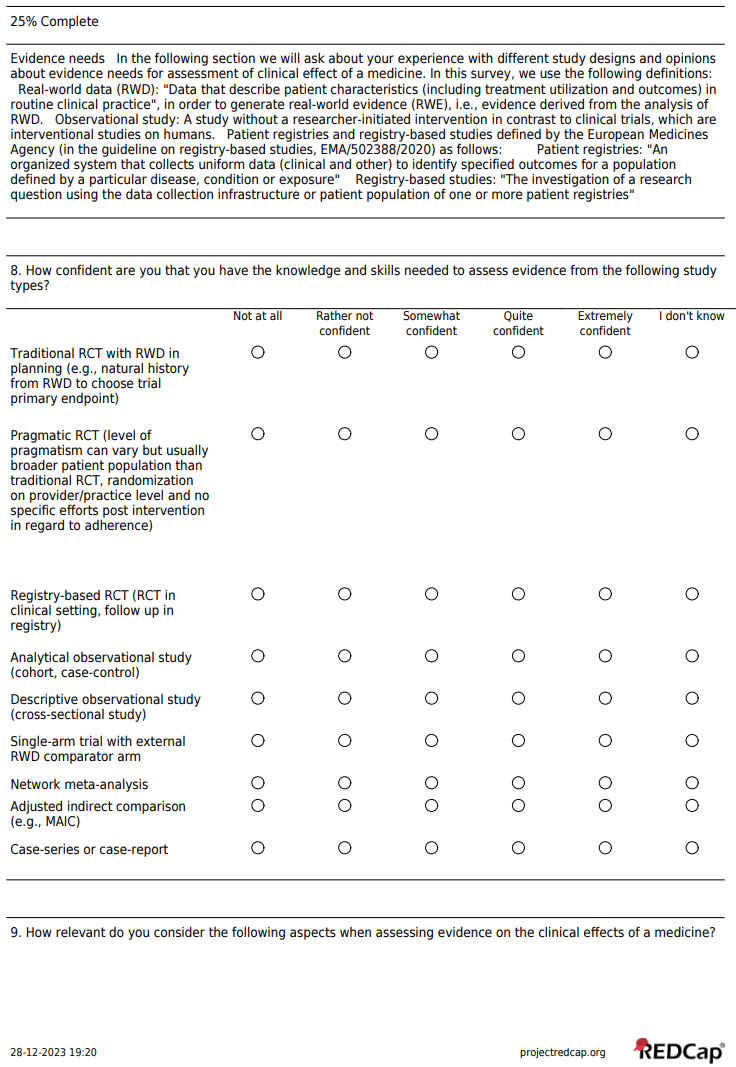

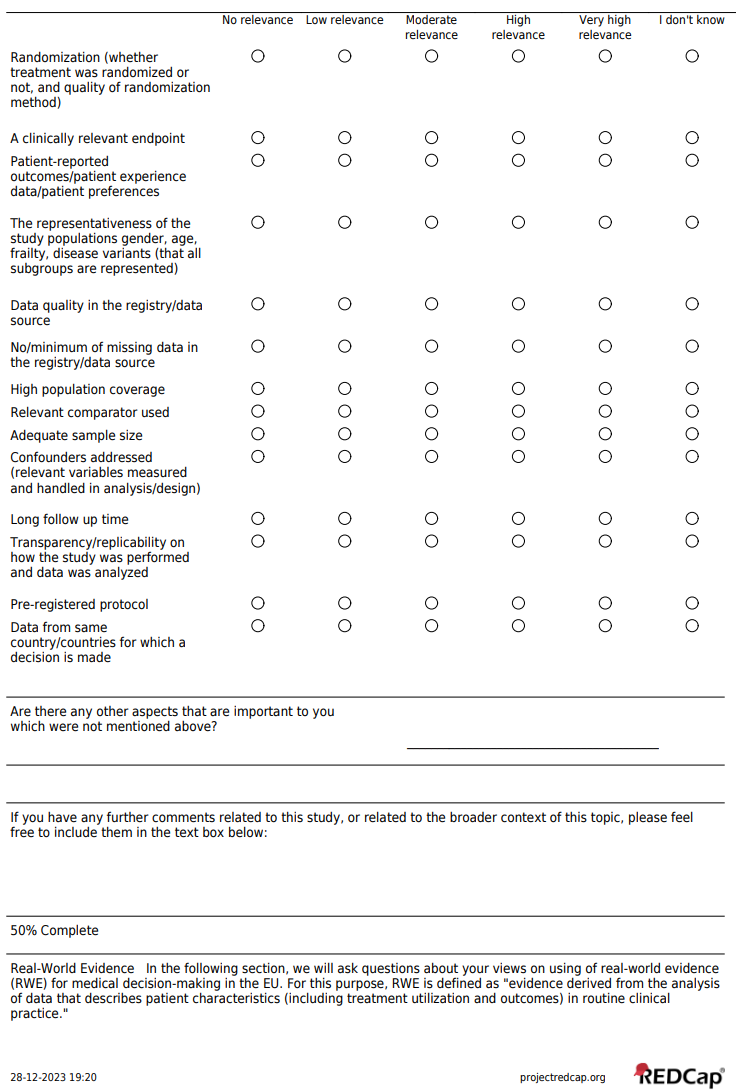

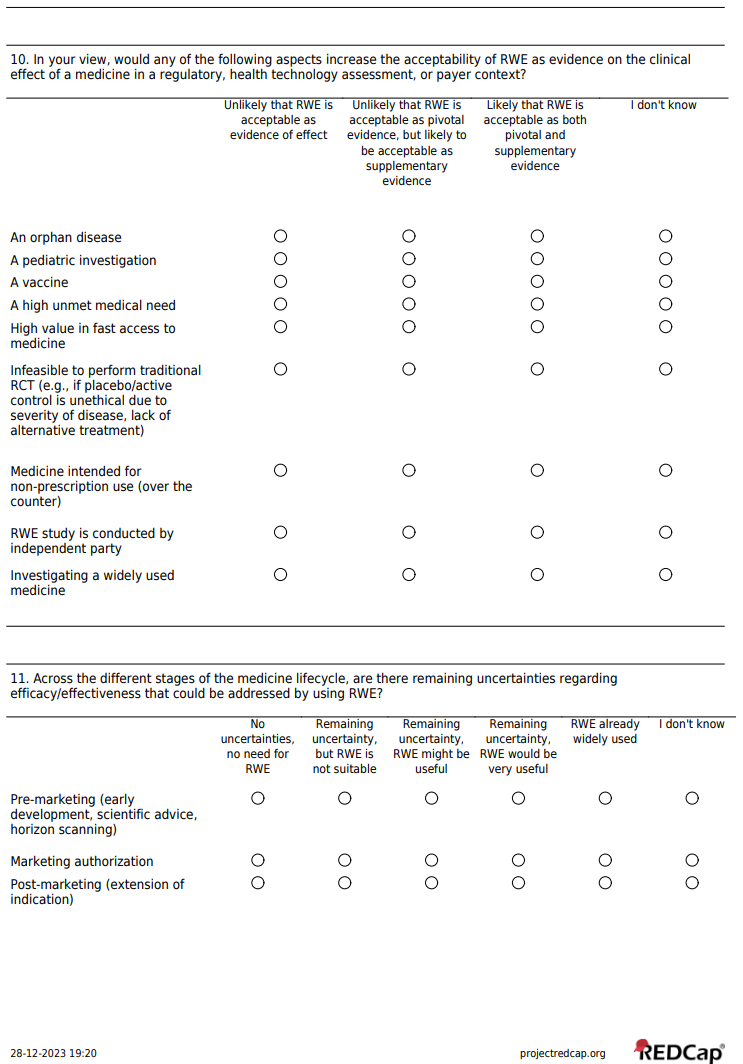

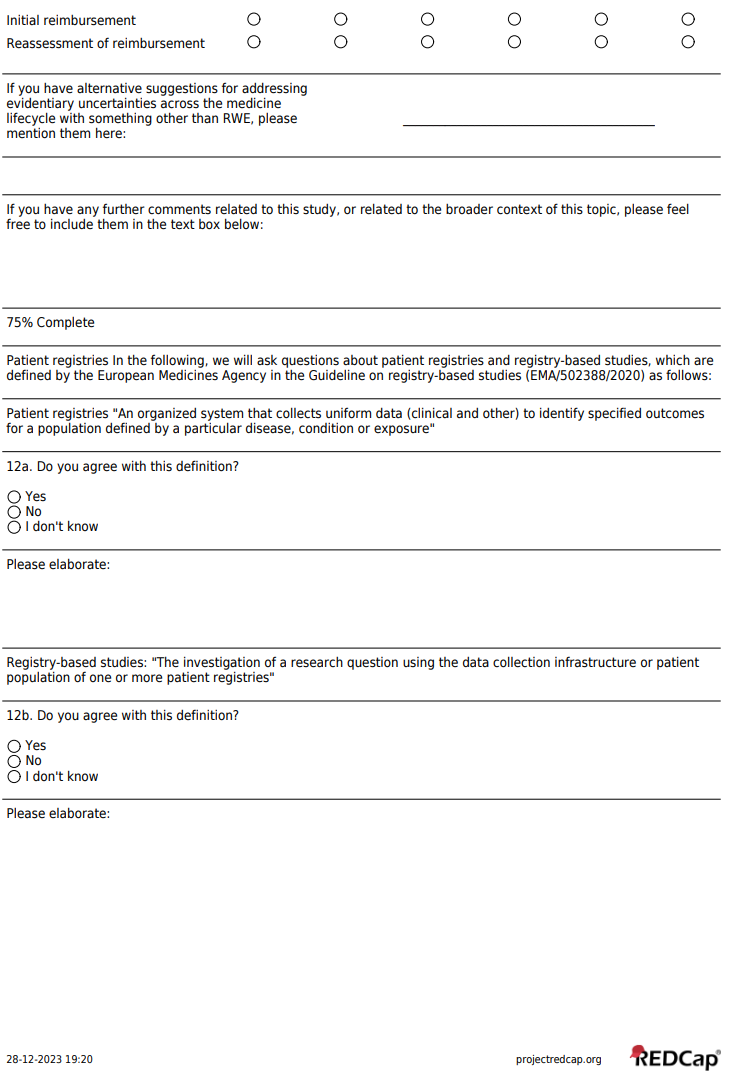

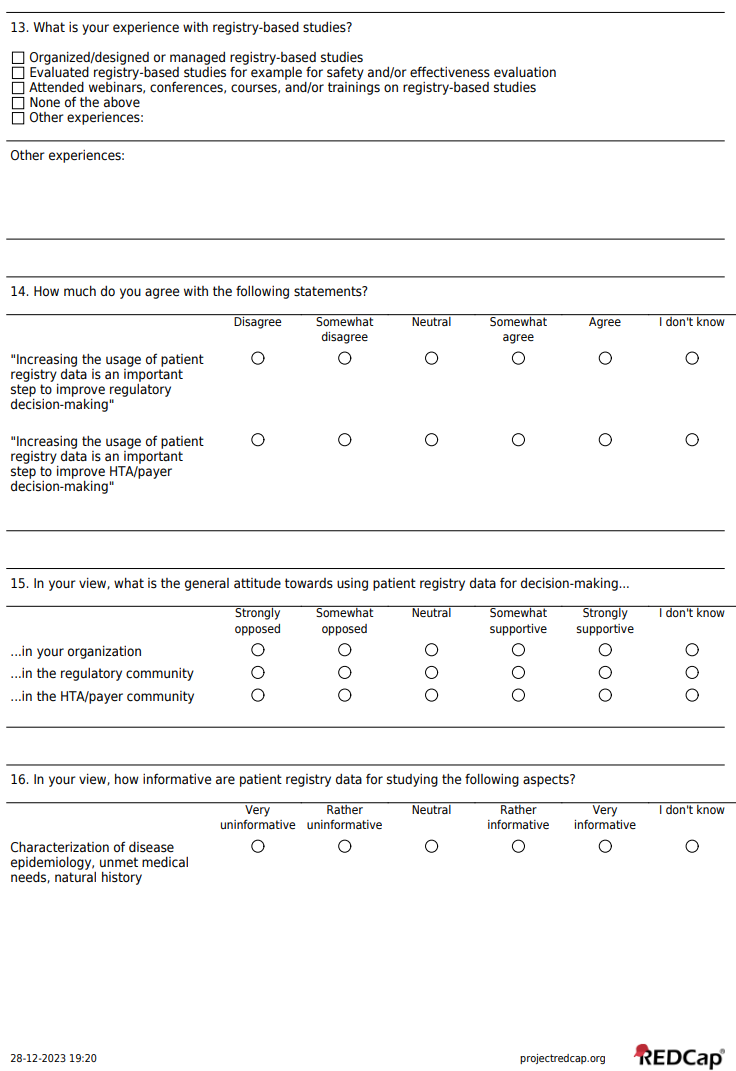

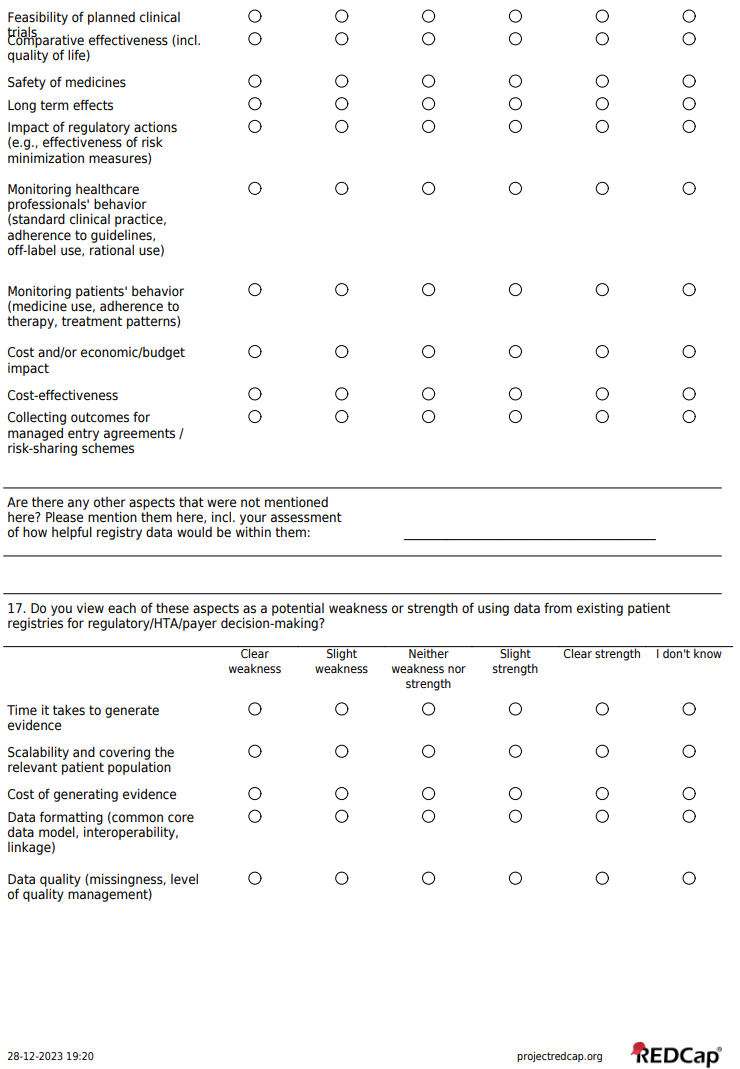

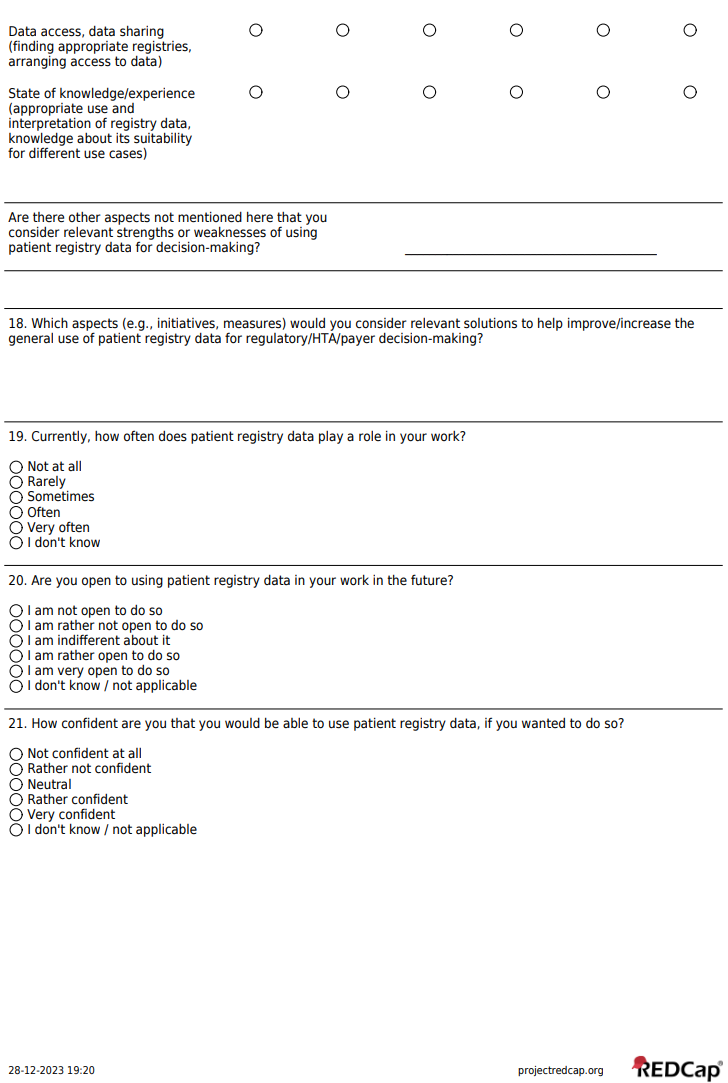

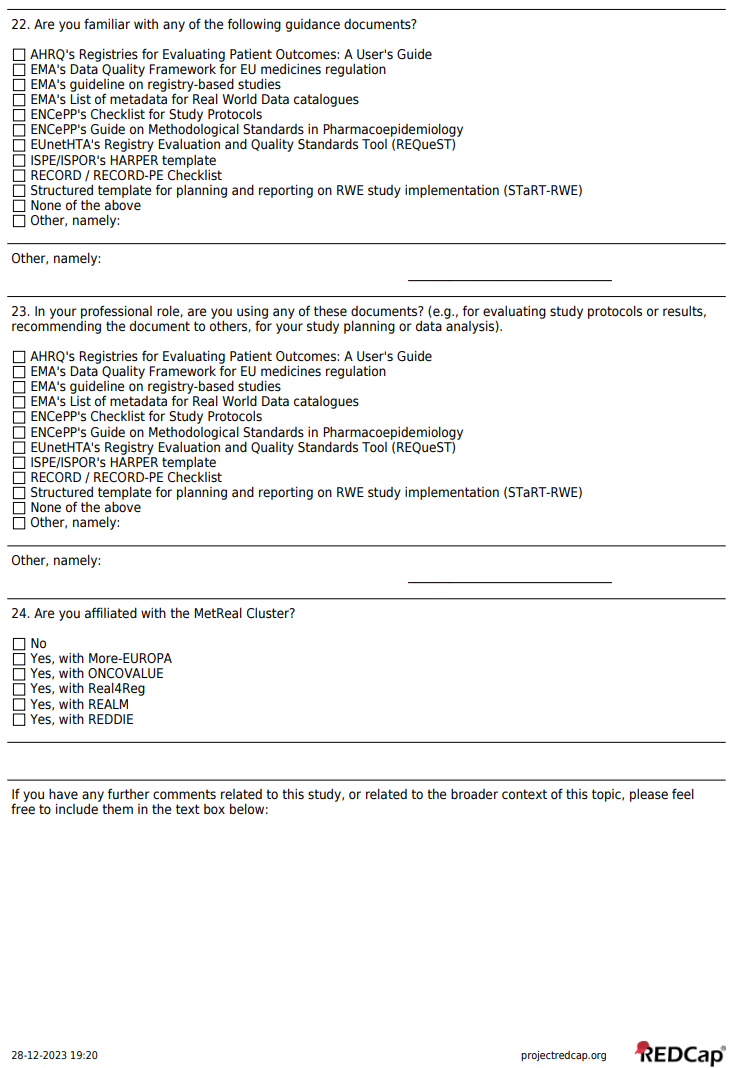


## Appendix C – Supplementary figures and tables

| Supplementary material 1A-D 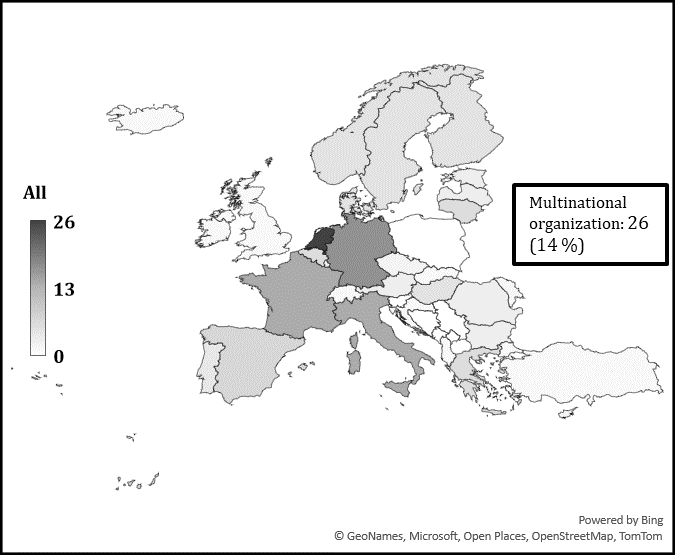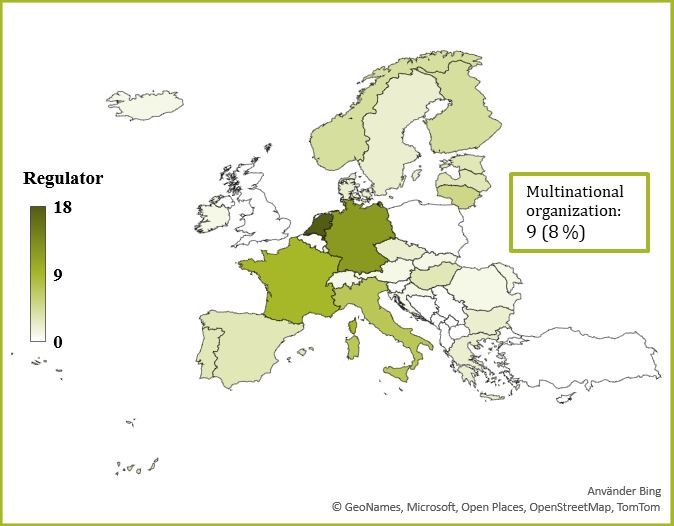  **B**  **A**  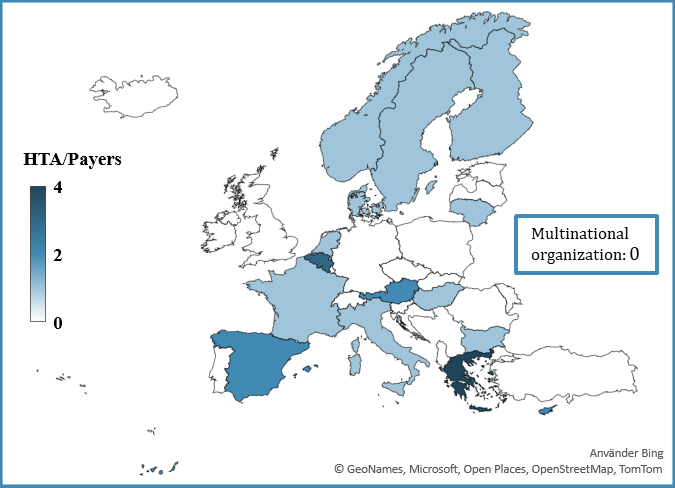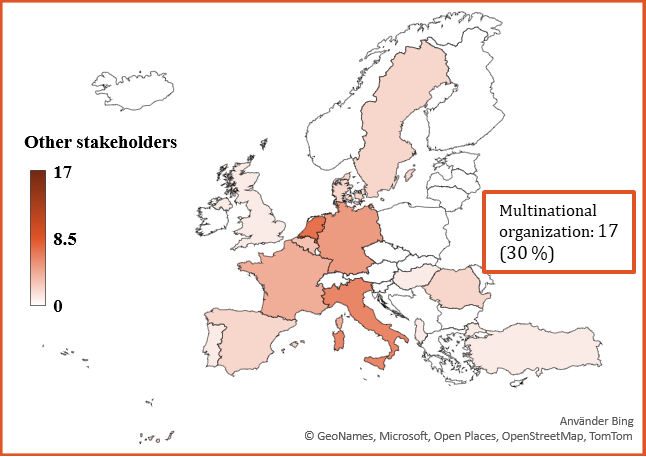  **D**  **C** |
| --- |
| *Participants' geographic distribution for all survey respondents (A), the regulators (B), HTA/Payers (C), and Other stakeholders (D). HTA = Health technology assessors* |

## Supplementary material 2: Respondents areas of involvement

| **A) Stages of involvement during the lifecycle of a medicinal product** | | | | | | | | | |
| --- | --- | --- | --- | --- | --- | --- | --- | --- | --- |
|  | **All** | **% of group** | **Regulators** | **% of group** | **HTA/ Payers** | **% of group** | **Other stakeholders** | | **% of group** |
| *Pre-clinical research/horizon scanning* | 26 | 14% | 9 | 8% | 9 | 38% | 8 | | 14% |
| *SA* | 82 | 43% | 70 | 64% | 8 | 33% | 4 | | 7% |
| *Initial MA*** | 92 | 48% | 88 | 80% | 3 | 13% | 1 | | 2% |
| *Renewal of MA / reassessment of conditional approvals *** | 70 | 37% | 67 | 61% | 1 | 4% | 2 | | 4% |
| *Marketing authorizations** | 15 | 8% | N/A | - | N/A | - | 15 | | 26% |
| *Initial reimbursement*** | 24 | 13% | 8 | 7% | 15 | 63% | 1 | | 2% |
| *Reimbursement reassessment*** | 16 | 8% | 4 | 4% | 12 | 50% | N/A | | - |
| *Procurement*** | 4 | 2% | 1 | 1% | 3 | 13% | N/A | | - |
| *Pricing including RSA*** | 12 | 6% | 3 | 3% | 8 | 33% | 1 | | 2% |
| *Reimbursement/pricing** | 13 | 7% | N/A | - | N/A | - | 13 | | 23% |
| *Recommendations/guidelines* | 51 | 27% | 29 | 26% | 8 | 33% | 14 | | 25% |
| *Post-marketing safety monitoring*** | 38 | 20% | 34 | 31% | 1 | 4% | 3 | | 5% |
| *Post-marketing research/PV** | 25 | 13% | N/A | - | N/A | - | 25 | | 44% |
| *Withdrawal*** | 41 | 21% | 32 | 29% | 8 | 33% | 1 | | 2% |
| *CD (phase 1)** | 8 | 4% | N/A | - | N/A | - | 8 | | 14% |
| *CD (phase 2)** | 13 | 7% | N/A | - | N/A | - | 13 | | 23% |
| *CD (phase 3)** | 16 | 8% | N/A | - | N/A | - | 16 | | 28% |
| *Communications (e.g., patient education, medical writing)** | 13 | 7% | N/A | - | N/A | - | 13 | | 23% |
| *None of the above* | 6 | 3% | 2 | 2% | 3 | 13% | 1 | | 2% |
| *Other* | 22 | 12% | 16 | 15% | 3 | 13% | 3 | | 5% |
| **B) Areas that respondents were involved in decisions in** | | | | | | | | | |
|  | **All** | **% of group** | **Regulators** | **% of group** | **HTA/ Payers** | **% of group** | **Other stakeholders** | | **% of group** |
| *All therapeutic areas / No specific therapeutic area* | 94 | 49% | 47 | 43% | 19 | 79% | 28 | | 49% |
| *Oncology and/or radiopharmaceuticals* | 45 | 24% | 30 | 27% | 4 | 17% | 11 | | 19% |
| *Vaccines* | 8 | 4% | 7 | 6% | 0 | 0% | 1 | | 2% |
| *Infectious diseases* | 14 | 7% | 10 | 9% | 1 | 4% | 3 | | 5% |
| *Neurological and/or psychiatric disorders* | 23 | 12% | 12 | 11% | 1 | 4% | 10 | | 18% |
| *Endocrine and/or cardiovascular diseases* | 9 | 5% | 2 | 2% | 2 | 8% | 5 | | 9% |
| *Immune and/or inflammatory diseases* | 23 | 12% | 12 | 11% | 2 | 8% | 9 | | 16% |
| *Advanced therapies (ATMPs)* | 27 | 14% | 17 | 15% | 3 | 13% | 7 | | 12% |
| *Hematological diseases* | 28 | 15% | 18 | 16% | 3 | 13% | 7 | | 12% |
| *Orphan medicines* | 38 | 20% | 27 | 25% | 2 | 8% | 9 | | 16% |
| *Pediatric medicines* | 24 | 13% | 16 | 15% | 2 | 8% | 6 | | 11% |
| *None of the above* | 3 | 2% | 2 | 2% | 0 | 0% | 1 | | 2% |
| *Other* | 11 | 6% | 7 | 6% | 0 | 0% | 4 | | 7% |
| *=not included in the survey for regulators and HTAs/payers, only for others  **= only included in survey for regulators and HTAs/payers, not for others  ***=merged Horizon scanning/prioritization for future assessment in survey of regulators and HTAs/payers and "fundamental or pre-clinical research/horizon scanning" in others survey  *ATMP: Advanced therapeutic medicinal product*  *HTA: Health technology assessors*  *SA: Scientific advice*  *CD: Clinical development*  *MA:* *Marketing authorizations*  *PV: Pharmacovigilance*  *RSA: Risk sharing agreements* | | | | | | | |  |  |

## Supplementary material 3: Number of responses per alternative for “having the knowledge and skills needed to assess evidence from the following study types” on a scale from 1 not at all to 5 extremely confident, including “I don´t know” and number of respondents in each group who did not respond to the question (blank).

|  |  | Not at all | Rather not confident | Somewhat confident | Quite confident | Extremely confident | I don't know | Blank |
| --- | --- | --- | --- | --- | --- | --- | --- | --- |
| RCT with RWD | All | 7 | 28 | 40 | 73 | 35 | 5 | 3 |
|  | Regulators | 3 | 13 | 21 | 46 | 23 | 2 | 2 |
|  | HTA/payers | 0 | 4 | 5 | 11 | 2 | 1 | 1 |
|  | Others | 4 | 11 | 14 | 16 | 10 | 2 | 0 |
| Pragmatic RCT | All | 9 | 44 | 47 | 61 | 20 | 7 | 3 |
|  | Regulators | 4 | 25 | 30 | 35 | 13 | 2 | 1 |
|  | HTA/payers | 1 | 4 | 6 | 8 | 1 | 2 | 1 |
|  | Others | 4 | 15 | 11 | 17 | 6 | 3 | 1 |
| Registry-based RCT | All | 7 | 41 | 53 | 63 | 18 | 6 | 3 |
|  | Regulators | 2 | 22 | 38 | 37 | 8 | 2 | 1 |
|  | HTA/payers | 0 | 6 | 6 | 7 | 1 | 2 | 1 |
|  | Others | 4 | 13 | 9 | 19 | 9 | 2 | 1 |
| Analytical observational study | All | 4 | 33 | 47 | 72 | 28 | 5 | 2 |
|  | Regulators | 4 | 21 | 29 | 45 | 8 | 2 | 1 |
|  | HTA/payers | 0 | 5 | 3 | 12 | 1 | 1 | 1 |
|  | Others | 0 | 7 | 14 | 15 | 19 | 2 | 0 |
| Descriptive observational study | All | 4 | 32 | 51 | 61 | 36 | 4 | 3 |
|  | Regulators | 3 | 22 | 32 | 40 | 11 | 1 | 1 |
|  | HTA/payers | 0 | 4 | 4 | 10 | 2 | 1 | 2 |
|  | Others | 0 | 6 | 15 | 11 | 23 | 2 | 0 |
| Single-arm trial with external RWD comparator | All | 7 | 48 | 62 | 53 | 13 | 5 | 3 |
|  | Regulators | 2 | 29 | 34 | 36 | 7 | 1 | 1 |
|  | HTA/payers | 1 | 4 | 8 | 6 | 0 | 2 | 2 |
|  | Others | 3 | 15 | 20 | 11 | 6 | 2 | 0 |
| Network meta-analyses | All | 16 | 68 | 53 | 39 | 6 | 7 | 2 |
|  | Regulators | 8 | 48 | 31 | 18 | 1 | 3 | 1 |
|  | HTA/payers | 1 | 3 | 4 | 13 | 0 | 1 | 1 |
|  | Others | 7 | 17 | 18 | 7 | 5 | 3 | 0 |
| Adjusted indirect comparison | All | 23 | 79 | 43 | 23 | 3 | 15 | 5 |
|  | Regulators | 11 | 54 | 23 | 12 | 1 | 6 | 3 |
|  | HTA/payers | 3 | 4 | 8 | 5 | 0 | 2 | 1 |
|  | Others | 9 | 21 | 11 | 6 | 2 | 7 | 1 |
| Case-series/case-report | All | 18 | 33 | 45 | 62 | 22 | 8 | 3 |
|  | Regulators | 6 | 24 | 27 | 34 | 13 | 4 | 2 |
|  | HTA/payers | 3 | 2 | 3 | 12 | 1 | 1 | 1 |
|  | Others | 8 | 7 | 15 | 16 | 8 | 3 | 0 |

*RCT = randomized controlled trial; RWD = real-world data; HTA = health technology assessors.*

## Supplementary material 4: Descriptive statistics. Mean and 95% confidence intervals (CI). Median and interquartial range (IQR), overall and per stakeholder group. N is number of responses that are not missing data or “I don’t know” Q10= If the aspects would increase acceptability of RWE, 1=unlikely that RWE is acceptable as evidence of effect, 2= Unlikely that RWE is acceptable as pivotal evidence, but likely to be acceptable as supplementary evidence, 3= Likely that RWE is acceptable as both pivotal and supplementary evidence. Q9= how relevant the attributes were for assessing evidence on clinical effect on a scale of 1, No relevance to 5, very high relevance. Q8= “having the knowledge and skills needed to assess evidence from the following study types” on a scale from 1 not at all to 5 extremely confident. Q11= “Across the different stages of the medicine lifecycle, are there remaining uncertainties regarding efficacy/Effectiveness that could be addressed by using RWE?” 1= No uncertainties, no need for RWE, 2= Remaining uncertainty, but RWE is not suitable, 3= Remining uncertainty, RWE might be useful, 4= Remining ucertainy, RWE would be very useful, 5=RWE already widely used.

*RCT = randomized controlled trial; RWD = real-world data; HTA = health technology assessors.*

| **Stakeholder group** | **Statement** | **N** | **median** | **IQR_ lower** | **IQR_ upper** | **mean** | **sd** | **ci_lower** | **ci_upper** |
| --- | --- | --- | --- | --- | --- | --- | --- | --- | --- |
| ***Results regarding “having the knowledge and skills needed to assess evidence from the following study types” on a scale from 1 not at all to 5 extremely confident.*** | | | | | | | | | |
| HTA/payers | RCT with RWD | 22 | 4 | 3 | 4 | 3.50 | 0.91 | 3.10 | 3.90 |
| Others | RCT with RWD | 55 | 3 | 2 | 4 | 3.31 | 1.20 | 2.98 | 3.63 |
| Overall | RCT with RWD | 183 | 4 | 3 | 4 | 3.55 | 1.08 | 3.39 | 3.71 |
| Regulators | RCT with RWD | 106 | 4 | 3 | 4 | 3.69 | 1.04 | 3.49 | 3.89 |
| HTA/payers | Pragmatic RCT | 21 | 3 | 3 | 4 | 3.24 | 1.00 | 2.79 | 3.69 |
| Others | Pragmatic RCT | 53 | 3 | 2 | 4 | 3.11 | 1.17 | 2.79 | 3.44 |
| Overall | Pragmatic RCT | 181 | 3 | 2 | 4 | 3.22 | 1.09 | 3.06 | 3.37 |
| Regulators | Pragmatic RCT | 107 | 3 | 2 | 4 | 3.26 | 1.07 | 3.06 | 3.47 |
| HTA/payers | Registry-based RCT | 21 | 3 | 2 | 4 | 3.05 | 1.02 | 2.58 | 3.51 |
| Others | Registry-based RCT | 54 | 4 | 2 | 4 | 3.30 | 1.22 | 2.96 | 3.63 |
| Overall | Registry-based RCT | 182 | 3 | 2 | 4 | 3.24 | 1.03 | 3.09 | 3.39 |
| Regulators | Registry-based RCT | 107 | 3 | 3 | 4 | 3.25 | 0.93 | 3.07 | 3.43 |
| HTA/payers | Analytical observational study | 22 | 4 | 3 | 4 | 3.41 | 0.91 | 3.01 | 3.81 |
| Others | Analytical observational study | 55 | 4 | 3 | 5 | 3.84 | 1.05 | 3.55 | 4.12 |
| Overall | Analytical observational study | 184 | 4 | 3 | 4 | 3.47 | 1.02 | 3.32 | 3.62 |
| Regulators | Analytical observational study | 107 | 3 | 3 | 4 | 3.30 | 0.99 | 3.11 | 3.49 |
| HTA/payers | Descriptive observational study | 21 | 4 | 3 | 4 | 3.38 | 1.07 | 2.89 | 3.87 |
| Others | Descriptive observational study | 55 | 4 | 3 | 5 | 3.93 | 1.07 | 3.64 | 4.22 |
| Overall | Descriptive observational study | 184 | 4 | 3 | 4 | 3.51 | 1.06 | 3.35 | 3.66 |
| Regulators | Descriptive observational study | 108 | 3 | 3 | 4 | 3.31 | 1.00 | 3.12 | 3.51 |
| HTA/payers | Single-arm trial with external RWD comparator arm | 20 | 3 | 2 | 4 | 2.90 | 0.97 | 2.45 | 3.35 |
| Others | Single-arm trial with external RWD comparator arm | 55 | 3 | 2 | 4 | 3.04 | 1.07 | 2.75 | 3.33 |
| Overall | Single-arm trial with external RWD comparator arm | 183 | 3 | 2 | 4 | 3.09 | 0.99 | 2.95 | 3.24 |
| Regulators | Single-arm trial with external RWD comparator arm | 108 | 3 | 2 | 4 | 3.16 | 0.96 | 2.97 | 3.34 |
| HTA/payers | Network meta-analyses | 22 | 4 | 3 | 4 | 3.41 | 0.91 | 3.01 | 3.81 |
| Others | Network meta-analyses | 54 | 3 | 2 | 3 | 2.74 | 1.14 | 2.43 | 3.05 |
| Overall | Network meta-analyses | 182 | 3 | 2 | 3 | 2.73 | 1.00 | 2.58 | 2.88 |
| Regulators | Network meta-analyses | 106 | 2 | 2 | 3 | 2.58 | 0.89 | 2.41 | 2.76 |
| HTA/payers | Adjusted indirect comparison | 21 | 3 | 2 | 3 | 2.76 | 1.00 | 2.31 | 3.21 |
| Others | Adjusted indirect comparison | 49 | 2 | 2 | 3 | 2.41 | 1.06 | 2.10 | 2.71 |
| Overall | Adjusted indirect comparison | 171 | 2 | 2 | 3 | 2.44 | 0.95 | 2.30 | 2.58 |
| Regulators | Adjusted indirect comparison | 101 | 2 | 2 | 3 | 2.39 | 0.87 | 2.21 | 2.56 |
| HTA/payers | Case-series/case-report | 22 | 4 | 2.25 | 4 | 3.18 | 1.26 | 2.62 | 3.74 |
| Others | Case-series/case-report | 54 | 3 | 2 | 4 | 3.17 | 1.27 | 2.82 | 3.51 |
| Overall | Case-series/case-report | 180 | 3 | 2 | 4 | 3.21 | 1.18 | 3.03 | 3.38 |
| Regulators | Case-series/case-report | 104 | 3 | 2 | 4 | 3.23 | 1.12 | 3.01 | 3.45 |
| ***Results regarding how relevant the attributes were for assessing evidence on clinical effect on a scale of 1, No relevance to 5, very high relevance.*** | | | | | | | | | |
| HTA/payers | Randomization | 24 | 5 | 4 | 5 | 4.58 | 0.78 | 4.26 | 4.91 |
| Others | Randomization | 55 | 4 | 4 | 5 | 4.36 | 0.73 | 4.17 | 4.56 |
| Overall | Randomization | 183 | 5 | 4 | 5 | 4.57 | 0.67 | 4.48 | 4.67 |
| Regulators | Randomization | 104 | 5 | 4 | 5 | 4.68 | 0.58 | 4.57 | 4.80 |
| HTA/payers | Confounders addressed | 24 | 4 | 4 | 5 | 4.25 | 0.61 | 3.99 | 4.51 |
| Others | Confounders addressed | 51 | 4 | 4 | 5 | 4.41 | 0.64 | 4.23 | 4.59 |
| Overall | Confounders addressed | 175 | 4 | 4 | 5 | 4.31 | 0.65 | 4.21 | 4.41 |
| Regulators | Confounders addressed | 100 | 4 | 4 | 5 | 4.27 | 0.66 | 4.14 | 4.40 |
| HTA/payers | Long follow up time | 24 | 4 | 4 | 5 | 4.21 | 0.59 | 3.96 | 4.46 |
| Others | Long follow up time | 54 | 4 | 3 | 4 | 3.87 | 0.78 | 3.66 | 4.08 |
| Overall | Long follow up time | 182 | 4 | 3 | 4 | 3.85 | 0.75 | 3.74 | 3.96 |
| Regulators | Long follow up time | 104 | 4 | 3 | 4 | 3.76 | 0.74 | 3.61 | 3.90 |
| HTA/payers | Transparency | 24 | 4 | 4 | 5 | 4.42 | 0.50 | 4.20 | 4.63 |
| Others | Transparency | 54 | 4 | 4 | 5 | 4.44 | 0.57 | 4.29 | 4.60 |
| Overall | Transparency | 182 | 4 | 4 | 5 | 4.42 | 0.62 | 4.33 | 4.51 |
| Regulators | Transparency | 104 | 5 | 4 | 5 | 4.40 | 0.68 | 4.27 | 4.54 |
| HTA/payers | Pre-registered protocol | 22 | 4 | 4 | 4 | 4.00 | 0.76 | 3.66 | 4.34 |
| Others | Pre-registered protocol | 51 | 4 | 3 | 4 | 3.88 | 0.82 | 3.65 | 4.11 |
| Overall | Pre-registered protocol | 173 | 4 | 4 | 5 | 4.11 | 0.82 | 3.99 | 4.23 |
| Regulators | Pre-registered protocol | 100 | 4 | 4 | 5 | 4.25 | 0.82 | 4.09 | 4.41 |
| HTA/payers | Country-specific decision data | 24 | 3 | 2.75 | 4 | 3.25 | 0.94 | 2.85 | 3.65 |
| Others | Country-specific decision data | 53 | 3 | 3 | 4 | 3.25 | 0.90 | 3.00 | 3.49 |
| Overall | Country-specific decision data | 180 | 3 | 3 | 4 | 3.11 | 0.86 | 2.98 | 3.24 |
| Regulators | Country-specific decision data | 103 | 3 | 3 | 3 | 3.01 | 0.82 | 2.85 | 3.17 |
| HTA/payers | A clinically relevant endpoint | 24 | 5 | 4.75 | 5 | 4.71 | 0.55 | 4.48 | 4.94 |
| Others | A clinically relevant endpoint | 54 | 5 | 4 | 5 | 4.67 | 0.51 | 4.53 | 4.81 |
| Overall | A clinically relevant endpoint | 184 | 5 | 5 | 5 | 4.72 | 0.55 | 4.64 | 4.80 |
| Regulators | A clinically relevant endpoint | 106 | 5 | 5 | 5 | 4.75 | 0.57 | 4.64 | 4.86 |
| HTA/payers | Patient preferences | 23 | 4 | 3 | 4 | 3.70 | 0.82 | 3.34 | 4.05 |
| Others | Patient preferences | 53 | 4 | 3 | 5 | 3.96 | 0.83 | 3.73 | 4.19 |
| Overall | Patient preferences | 183 | 4 | 3 | 4 | 3.72 | 0.81 | 3.60 | 3.83 |
| Regulators | Patient preferences | 107 | 3 | 3 | 4 | 3.60 | 0.78 | 3.45 | 3.75 |
| HTA/payers | Population demographics and variability | 24 | 4 | 4 | 5 | 4.17 | 0.82 | 3.82 | 4.51 |
| Others | Population demographics and variability | 55 | 4 | 4 | 5 | 4.18 | 0.67 | 4.00 | 4.36 |
| Overall | Population demographics and variability | 186 | 4 | 4 | 5 | 4.19 | 0.72 | 4.09 | 4.30 |
| Regulators | Population demographics and variability | 107 | 4 | 4 | 5 | 4.21 | 0.72 | 4.07 | 4.34 |
| HTA/payers | Data source | 24 | 4 | 4 | 5 | 4.33 | 0.76 | 4.01 | 4.65 |
| Others | Data source | 55 | 5 | 4 | 5 | 4.55 | 0.66 | 4.37 | 4.72 |
| Overall | Data source | 185 | 5 | 4 | 5 | 4.50 | 0.64 | 4.40 | 4.59 |
| Regulators | Data source | 106 | 5 | 4 | 5 | 4.51 | 0.61 | 4.39 | 4.63 |
| HTA/payers | Minimal missing data in registry | 23 | 4 | 4 | 4 | 3.87 | 0.69 | 3.57 | 4.17 |
| Others | Minimal missing data in registry | 54 | 4 | 3 | 4 | 3.76 | 0.80 | 3.54 | 3.98 |
| Overall | Minimal missing data in registry | 180 | 4 | 3 | 4 | 3.81 | 0.78 | 3.69 | 3.92 |
| Regulators | Minimal missing data in registry | 103 | 4 | 3 | 4 | 3.82 | 0.80 | 3.66 | 3.97 |
| HTA/payers | High population coverage | 24 | 4 | 4 | 4.25 | 4.00 | 0.78 | 3.67 | 4.33 |
| Others | High population coverage | 51 | 4 | 3 | 4 | 3.86 | 0.78 | 3.64 | 4.08 |
| Overall | High population coverage | 181 | 4 | 3 | 4 | 3.82 | 0.76 | 3.71 | 3.93 |
| Regulators | High population coverage | 106 | 4 | 3 | 4 | 3.76 | 0.75 | 3.62 | 3.91 |
| HTA/payers | Relevant comparator used | 24 | 5 | 4 | 5 | 4.54 | 0.51 | 4.33 | 4.76 |
| Others | Relevant comparator used | 53 | 4 | 4 | 5 | 4.30 | 0.70 | 4.11 | 4.49 |
| Overall | Relevant comparator used | 182 | 4 | 4 | 5 | 4.39 | 0.69 | 4.29 | 4.49 |
| Regulators | Relevant comparator used | 105 | 4 | 4 | 5 | 4.40 | 0.72 | 4.26 | 4.54 |
| HTA/payers | Adequate sample size | 23 | 4 | 4 | 5 | 4.39 | 0.50 | 4.18 | 4.61 |
| Others | Adequate sample size | 53 | 4 | 4 | 5 | 4.32 | 0.75 | 4.11 | 4.53 |
| Overall | Adequate sample size | 182 | 4 | 4 | 5 | 4.31 | 0.69 | 4.21 | 4.41 |
| Regulators | Adequate sample size | 106 | 4 | 4 | 5 | 4.28 | 0.70 | 4.15 | 4.42 |

## Supplementary material 5: Results of differences across stakeholder groups

*RCT = randomized controlled trial; RWD = real-world data; HTA = health technology assessors. df=degrees of freedom*

| **Statement** | **H** | **df** | **p_value** |  |
| --- | --- | --- | --- | --- |
| ***Results regarding “having the knowledge and skills needed to assess evidence from the following study types” on a scale from 1 not at all to 5 extremely confident.*** | | | | |
| RCT with RWD | 4.207 | 2 | 0.122 |  |
| Pragmatic RCT | 0.542 | 2 | 0.762 |  |
| Registry-based RCT | 1.005 | 2 | 0.605 |  |
| Single-arm trial with external RWD comparator arm | 1.156 | 2 | 0.561 |  |
| Adjusted indirect comparison | 3.691 | 2 | 0.158 |  |
| Case-series/case-report | 0.055 | 2 | 0.973 |  |
| Analytical observational study | 9.414 | 2 | 0.009 | * |
| Descriptive observational study | 11.891 | 2 | 0.003 | * |
| Network meta-analyses | 13.331 | 2 | 0.001 | * |
| ***Results regarding how relevant the attributes were for assessing evidence on clinical effect on a scale of 1, No relevance to 5, very high relevance.*** | | | | |
| A clinically relevant endpoint | 2.161 | 2 | 0.339 |  |
| Population demographics and variability | 0.112 | 2 | 0.946 |  |
| Data source | 1.777 | 2 | 0.411 |  |
| Minimal missing data in registry | 0.603 | 2 | 0.740 |  |
| High population coverage | 2.390 | 2 | 0.303 |  |
| Relevant comparator used | 1.952 | 2 | 0.377 |  |
| Adequate sample size | 0.296 | 2 | 0.863 |  |
| Confounders addressed | 1.995 | 2 | 0.369 |  |
| Transparency | 0.108 | 2 | 0.947 |  |
| Country-specific decision data | 2.912 | 2 | 0.233 |  |
| Randomization | 10.421 | 2 | 0.005 | * |
| Patient preferences | 6.364 | 2 | 0.041 | * |
| Long follow up time | 7.114 | 2 | 0.029 | * |
| Pre-registered protocol | 8.517 | 2 | 0.014 | * |

## Supplementary material 6: Results from post-hoc pairwise comparison of stakeholder groups

*p.unadj = unadjusted p-value. P.adj = p-value with Bonferroni correction***.** *RCT = randomized controlled trial; RWD = real-world data; HTA = health technology assessors.*

| **Statement** | **Comparison** | **Z** | **P.unadj** | **P.adj** |  | **r** |
| --- | --- | --- | --- | --- | --- | --- |
| ***Results regarding “having the knowledge and skills needed to assess evidence from the following study types” on a scale from 1 not at all to 5 extremely confident.*** | | | | | | |
| Analytical observational study | HTA/payers - Others | -1.605 | 0.108 | 0.325 |  | -0.118351466 |
| Analytical observational study | HTA/payers - Regulators | 0.434 | 0.665 | 1.000 |  | 0.031960392 |
| Analytical observational study | Others - Regulators | 3.053 | 0.002 | 0.007 | * | 0.225040009 |
| Descriptive observational study | HTA/payers - Others | -1.859 | 0.063 | 0.189 |  | -0.137074929 |
| Descriptive observational study | HTA/payers - Regulators | 0.376 | 0.707 | 1.000 |  | 0.027710074 |
| Descriptive observational study | Others - Regulators | 3.420 | 0.001 | 0.002 | * | 0.252156752 |
| Network meta-analyses | Others - Regulators | 0.790 | 0.430 | 1.000 |  | 0.058552819 |
| Network meta-analyses | HTA/payers - Others | 2.859 | 0.004 | 0.013 | * | 0.211908785 |
| Network meta-analyses | HTA/payers - Regulators | 3.650 | 0.000 | 0.001 | * | 0.270558783 |
| ***Results regarding how relevant the attributes were for assessing evidence on clinical effect on a scale of 1, No relevance to 5, very high relevance.*** | | | | | | |
| Long follow up time | HTA/payers - Others | 1.976 | 0.048 | 0.144 |  | 0.146472035 |
| Long follow up time | Others - Regulators | 0.711 | 0.477 | 1.000 |  | 0.052670124 |
| Long follow up time | HTA/payers - Regulators | 2.667 | 0.008 | 0.023 | * | 0.197689897 |
| Patient preferences | HTA/payers - Others | -1.179 | 0.238 | 0.715 |  | -0.087152005 |
| Patient preferences | HTA/payers - Regulators | 0.562 | 0.574 | 1.000 |  | 0.041580524 |
| Patient preferences | Others - Regulators | 2.522 | 0.012 | 0.035 | * | 0.186449602 |
| Pre-registered protocol | HTA/payers - Others | 0.532 | 0.595 | 1.000 |  | 0.040423007 |
| Pre-registered protocol | HTA/payers - Regulators | -1.474 | 0.141 | 0.422 |  | -0.112031908 |
| Pre-registered protocol | Others - Regulators | -2.805 | 0.005 | 0.015 | * | -0.213245406 |
| Randomization | HTA/payers - Others | 1.831 | 0.067 | 0.202 |  | 0.135316061 |
| Randomization | HTA/payers - Regulators | -0.376 | 0.707 | 1.000 |  | -0.027764899 |
| Randomization | Others - Regulators | -3.196 | 0.001 | 0.004 | * | -0.236264395 |

## Supplementary material 7: Distribution of responses in total (all) and per group for “having the knowledge and skills needed to assess evidence from the following study types” on a scale from 1 not at all to 5 extremely confident.

***
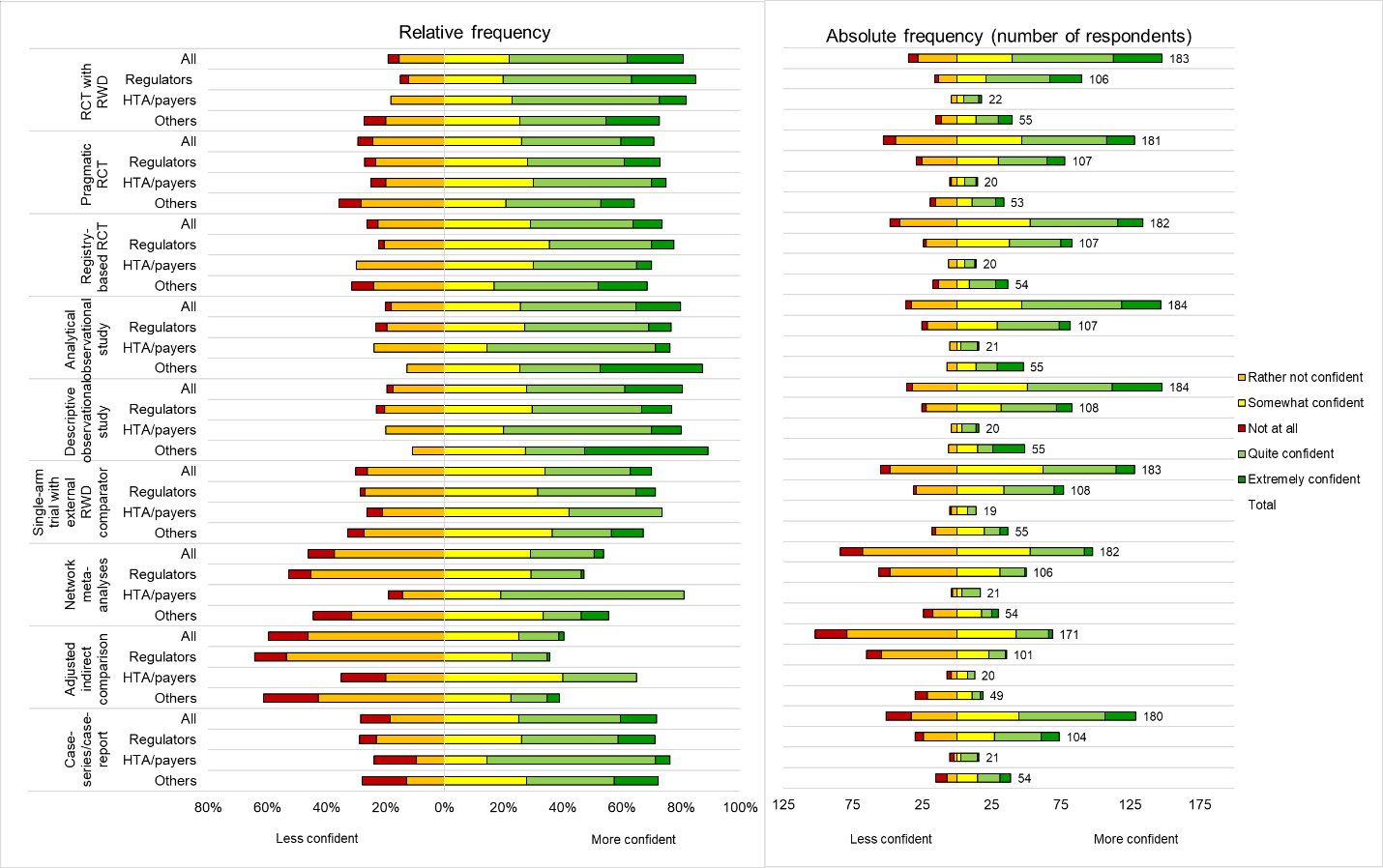
***

*RCT = randomized controlled trial; RWD = real-world data; HTA = health technology assessors.*

## Supplementary material 8: Number of responses regarding how relevant the attributes were for assessing evidence on clinical effect on a scale of 1, No relevance to 5, very high relevance.

|  |  | **No relevance** | **Low relevance** | **Moderate relevance** | **High relevance** | **Very high relevance** | **I don't know** | **Blank** |
| --- | --- | --- | --- | --- | --- | --- | --- | --- |
| Randomization | All | 0 | 3 | 9 | 51 | 120 | 4 | 4 |
|  | Regulators | 0 | 0 | 6 | 21 | 77 | 2 | 4 |
|  | HTA/payers | 0 | 1 | 1 | 5 | 17 | 0 | 0 |
|  | Others | 0 | 2 | 2 | 25 | 26 | 2 | 0 |
| Clinically relevant endpoint | All | 0 | 1 | 6 | 37 | 140 | 4 | 3 |
|  | Regulators | 0 | 1 | 4 | 16 | 85 | 2 | 2 |
|  | HTA/payers | 0 | 0 | 1 | 5 | 18 | 0 | 0 |
|  | Others | 0 | 0 | 1 | 16 | 37 | 2 | 1 |
| Patient preferences | All | 0 | 5 | 78 | 64 | 36 | 4 | 4 |
|  | Regulators | 0 | 4 | 50 | 38 | 15 | 2 | 1 |
|  | HTA/payers | 0 | 1 | 9 | 9 | 4 | 0 | 1 |
|  | Others | 0 | 0 | 19 | 17 | 17 | 2 | 2 |
| Population demographics and variability | All | 0 | 2 | 27 | 90 | 67 | 4 | 1 |
|  | Regulators | 0 | 1 | 16 | 50 | 40 | 2 | 1 |
|  | HTA/payers | 0 | 1 | 3 | 11 | 9 | 0 | 0 |
|  | Others | 0 | 0 | 8 | 29 | 18 | 2 | 0 |
| Data quality | All | 0 | 1 | 12 | 66 | 106 | 5 | 1 |
|  | Regulators | 0 | 0 | 6 | 40 | 60 | 3 | 1 |
|  | HTA/payers | 0 | 1 | 1 | 11 | 11 | 0 | 0 |
|  | Others | 0 | 0 | 5 | 15 | 35 | 2 | 0 |
| Minimal missing data in registry | All | 1 | 6 | 52 | 89 | 32 | 7 | 4 |
|  | Regulators | 1 | 3 | 29 | 51 | 19 | 4 | 3 |
|  | HTA/payers | 0 | 1 | 4 | 15 | 3 | 0 | 1 |
|  | Others | 0 | 2 | 19 | 23 | 10 | 3 | 0 |
| High population coverage | All | 0 | 7 | 50 | 92 | 32 | 8 | 2 |
|  | Regulators | 0 | 4 | 33 | 53 | 16 | 3 | 1 |
|  | HTA/payers | 0 | 1 | 4 | 13 | 6 | 0 | 0 |
|  | Others | 0 | 2 | 13 | 26 | 10 | 5 | 1 |
| Relevant comparator used | All | 1 | 2 | 9 | 83 | 87 | 7 | 2 |
|  | Regulators | 1 | 1 | 5 | 46 | 52 | 3 | 2 |
|  | HTA/payers | 0 | 0 | 0 | 11 | 13 | 0 | 0 |
|  | Others | 0 | 1 | 4 | 26 | 22 | 4 | 0 |
| Adequate sample size | All | 0 | 2 | 18 | 84 | 78 | 5 | 4 |
|  | Regulators | 0 | 2 | 9 | 52 | 43 | 2 | 2 |
|  | HTA/payers | 0 | 0 | 0 | 14 | 9 | 0 | 1 |
|  | Others | 0 | 0 | 9 | 18 | 26 | 3 | 1 |
| Confounders addressed | All | 0 | 1 | 15 | 88 | 71 | 12 | 4 |
|  | Regulators | 0 | 1 | 9 | 52 | 38 | 6 | 4 |
|  | HTA/payers | 0 | 0 | 2 | 14 | 8 | 0 | 0 |
|  | Others | 0 | 0 | 4 | 22 | 25 | 6 | 0 |
| Long follow up time | All | 0 | 3 | 57 | 86 | 36 | 6 | 3 |
|  | Regulators | 0 | 3 | 35 | 50 | 16 | 3 | 3 |
|  | HTA/payers | 0 | 0 | 2 | 15 | 7 | 0 | 0 |
|  | Others | 0 | 0 | 20 | 21 | 13 | 3 | 0 |
| Transparency | All | 0 | 0 | 13 | 80 | 89 | 5 | 4 |
|  | Regulators | 0 | 0 | 11 | 40 | 53 | 2 | 4 |
|  | HTA/payers | 0 | 0 | 0 | 14 | 10 | 0 | 0 |
|  | Others | 0 | 0 | 2 | 26 | 26 | 3 | 0 |
| Pre-registered protocol | All | 1 | 4 | 32 | 74 | 62 | 14 | 4 |
|  | Regulators | 1 | 1 | 15 | 38 | 45 | 6 | 4 |
|  | HTA/payers | 0 | 1 | 3 | 13 | 5 | 2 | 0 |
|  | Others | 0 | 2 | 14 | 23 | 12 | 6 | 0 |
| Country-specific decision data | All | 5 | 34 | 86 | 46 | 9 | 6 | 5 |
|  | Regulators | 4 | 19 | 55 | 22 | 3 | 2 | 5 |
|  | HTA/payers | 0 | 6 | 8 | 8 | 2 | 0 | 0 |
|  | Others | 1 | 9 | 23 | 16 | 4 | 4 | 0 |

*HTA = health technology assessors.*

## Supplementary material 9: Distribution of responses regarding how relevant the attributes were for assessing evidence on clinical effect on a scale of 1, No relevance to 5, very high relevance in relative and absolute frequencies, in total (“all”) and per group.

*
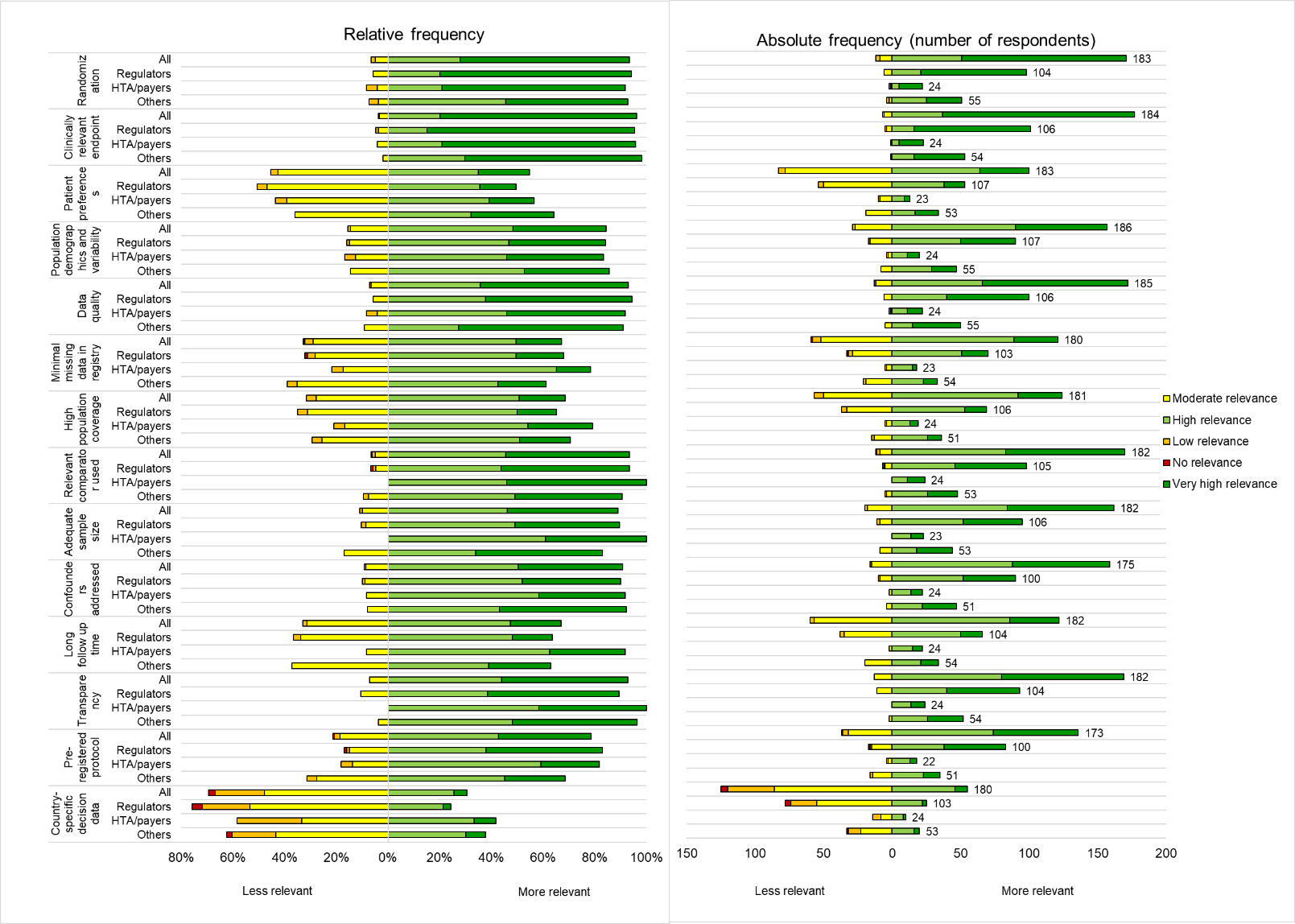
*

## Supplementary material 10: Respondents views on remaining uncertainties regarding efficacy/effectiveness that could be addressed by using RWE in different life cycle stages, absolute number of responses.

|  |  | No uncertainties, no need for RWE | Remaining uncertainty, but RWE is not suitable | Remaining uncertainty, RWE might be useful | Remaining uncertainty, RWE would be very useful | RWE already widely used | I don't know | Blank |
| --- | --- | --- | --- | --- | --- | --- | --- | --- |
| Pre-marketing | All | 12 | 41 | 52 | 37 | 12 | 29 | 8 |
|  | Regulators | 10 | 24 | 37 | 13 | 6 | 16 | 4 |
|  | HTA/payers | 1 | 6 | 6 | 4 | 0 | 4 | 3 |
|  | Others | 1 | 11 | 9 | 20 | 6 | 9 | 1 |
| Marketing authorization | All | 7 | 39 | 66 | 41 | 7 | 24 | 7 |
|  | Regulators | 7 | 25 | 45 | 17 | 0 | 13 | 3 |
|  | HTA/payers | 0 | 7 | 7 | 3 | 0 | 4 | 3 |
|  | Others | 0 | 7 | 14 | 21 | 7 | 7 | 1 |
| Post-marketing | All | 2 | 14 | 54 | 68 | 28 | 18 | 7 |
|  | Regulators | 2 | 10 | 45 | 29 | 11 | 10 | 3 |
|  | HTA/payers | 0 | 2 | 6 | 8 | 3 | 2 | 3 |
|  | Others | 0 | 2 | 3 | 31 | 14 | 6 | 1 |
| Initial reimbursement | All | 2 | 20 | 48 | 48 | 11 | 53 | 9 |
|  | Regulators | 2 | 10 | 28 | 19 | 4 | 43 | 4 |
|  | HTA/payers | 0 | 5 | 8 | 7 | 0 | 1 | 3 |
|  | Others | 0 | 5 | 12 | 22 | 7 | 9 | 2 |
| Reassessment of reimbursement | All | 0 | 5 | 24 | 71 | 31 | 53 | 7 |
|  | Regulators | 0 | 1 | 13 | 37 | 13 | 43 | 3 |
|  | HTA/payers | 0 | 1 | 5 | 9 | 6 | 0 | 3 |
|  | Others | 0 | 3 | 6 | 25 | 12 | 10 | 1 |

*RWE = real-world evidence; HTA = health technology assessors*

## Supplementary material 11: Respondents’ views on aspects of accepting RWE as evidence of the clinical effects of medicine in different situations, for all respondents and stratified per stakeholder group, absolute number of responses.

|  |  | Unlikely to accept RWE as evidence of effect | Unlikely to accept RWE as pivotal evidence, but likely to accept as supplementary evidence | Likely to accept RWE as both pivotal and supplementary evidence | I don't know | Blank |
| --- | --- | --- | --- | --- | --- | --- |
| **Infeasible to perform traditional RCT** | All | 7 | 62 | 101 | 14 | 7 |
|  | Regulators | 5 | 45 | 49 | 8 | 3 |
|  | HTA/payers | 1 | 8 | 12 | 0 | 3 |
|  | Others | 1 | 9 | 40 | 6 | 1 |
| **Orphan disease** | All | 4 | 77 | 86 | 15 | 9 |
|  | Regulators | 4 | 55 | 38 | 9 | 4 |
|  | HTA/payers | 0 | 11 | 10 | 0 | 3 |
|  | Others | 0 | 11 | 38 | 6 | 2 |
| **A high unmet clinical need** | All | 11 | 83 | 74 | 15 | 8 |
|  | Regulators | 6 | 59 | 32 | 9 | 4 |
|  | HTA/payers | 3 | 8 | 9 | 1 | 3 |
|  | Others | 2 | 16 | 33 | 5 | 1 |
| **Investigating a widely used medicine** | All | 22 | 79 | 61 | 21 | 8 |
|  | Regulators | 16 | 47 | 32 | 11 | 4 |
|  | HTA/payers | 3 | 9 | 8 | 1 | 3 |
|  | Others | 3 | 23 | 21 | 9 | 1 |
| **Pediatric investigation** | All | 11 | 99 | 57 | 16 | 8 |
|  | Regulators | 7 | 68 | 21 | 10 | 4 |
|  | HTA/payers | 2 | 9 | 10 | 0 | 3 |
|  | Others | 2 | 22 | 26 | 6 | 1 |
| **High value in fast access to medicine** | All | 22 | 86 | 45 | 23 | 15 |
|  | Regulators | 14 | 54 | 20 | 16 | 6 |
|  | HTA/payers | 5 | 10 | 5 | 0 | 4 |
|  | Others | 3 | 22 | 20 | 7 | 5 |
| **A vaccine** | All | 33 | 78 | 36 | 37 | 7 |
|  | Regulators | 21 | 50 | 13 | 23 | 3 |
|  | HTA/payers | 4 | 5 | 7 | 5 | 3 |
|  | Others | 8 | 23 | 16 | 9 | 1 |
| **RWE study conducted by independent party** | All | 15 | 97 | 31 | 38 | 10 |
|  | Regulators | 10 | 65 | 12 | 18 | 5 |
|  | HTA/payers | 1 | 10 | 5 | 4 | 4 |
|  | Others | 4 | 22 | 14 | 16 | 1 |
| **Medicine for non-prescription use** | All | 56 | 65 | 27 | 35 | 8 |
|  | Regulators | 41 | 39 | 11 | 15 | 4 |
|  | HTA/payers | 3 | 8 | 6 | 4 | 3 |
|  | Others | 12 | 18 | 10 | 16 | 1 |

*HTA = Health technology assessors; RWE = Real-world evidence, absolute no of responses*
